# Sex-Specific Patterns of Mortality Predictors Among Patients Undergoing Cardiac Resynchronization Therapy: A Machine Learning Approach

**DOI:** 10.3389/fcvm.2021.611055

**Published:** 2021-02-25

**Authors:** Márton Tokodi, Anett Behon, Eperke Dóra Merkel, Attila Kovács, Zoltán Tősér, András Sárkány, Máté Csákvári, Bálint Károly Lakatos, Walter Richard Schwertner, Annamária Kosztin, Béla Merkely

**Affiliations:** ^1^Heart and Vascular Center, Semmelweis University, Budapest, Hungary; ^2^Argus Cognitive, Inc., Lebanon, NH, United States

**Keywords:** heart failure, cardiac resynchronization therapy, sex differences, machine learning, mortality prediction

## Abstract

**Background:** The relative importance of variables explaining sex-related differences in outcomes is scarcely explored in patients undergoing cardiac resynchronization therapy (CRT). We sought to implement and evaluate machine learning (ML) algorithms for the prediction of 1- and 3-year all-cause mortality in CRT patients. We also aimed to assess the sex-specific differences in predictors of mortality utilizing ML.

**Methods:** Using a retrospective registry of 2,191 CRT patients, ML models were implemented in 6 partially overlapping patient subsets (all patients, females, or males with 1- or 3-year follow-up). Each cohort was randomly split into training (80%) and test sets (20%). After hyperparameter tuning in the training sets, the best performing algorithm was evaluated in the test sets. Model discrimination was quantified using the area under the receiver-operating characteristic curves (AUC). The most important predictors were identified using the permutation feature importances method.

**Results:** Conditional inference random forest exhibited the best performance with AUCs of 0.728 (0.645–0.802) and 0.732 (0.681–0.784) for the prediction of 1- and 3-year mortality, respectively. Etiology of heart failure, NYHA class, left ventricular ejection fraction, and QRS morphology had higher predictive power, whereas hemoglobin was less important in females compared to males. The importance of atrial fibrillation and age increased, while the importance of serum creatinine decreased from 1- to 3-year follow-up in both sexes.

**Conclusions:** Using ML techniques in combination with easily obtainable clinical features, our models effectively predicted 1- and 3-year all-cause mortality in CRT patients. Sex-specific patterns of predictors were identified, showing a dynamic variation over time.

## Introduction

Despite the comparable overall lifetime risk of heart failure (HF) between sexes (1, 2), there are notable differences between males and females with HF across the entire spectrum of ejection fraction (EF) (3). In HF patients with reduced EF (HFrEF), several studies have highlighted sex-related differences that involve multiple aspects of the syndrome, such as epidemiology, pathophysiology, phenotyping, and prognosis (4). Nevertheless, females are under-represented in HFrEF trials questioning their generalizability and leaving significant gaps in knowledge (4, 5).

While women with HFrEF have better survival and lower hospitalization rates, they have a greater burden of symptoms and more impaired health-related quality of life than men (6). Although sex disparities are also remarkable in the accessibility to HF device therapy, including cardiac resynchronization therapy (CRT) (7–9), women are more likely to respond favorably and derive a greater survival benefit from CRT implantation (10–13). Nonetheless, the sex-related differences in both short- and long-term outcomes and the varying importance of different predictors are still scarcely explored in this patient population (14). One conceivable explanation could be the failure of the applied statistical methods to harness the potential prognostic value of complex interactions between several weaker, often unexpected risk factors and the outcome. However, this limitation might be circumvented by advanced data analytic techniques (15).

To improve predictive modeling and elucidate novel determinants of a specific outcome, machine learning (ML) has been increasingly utilized in cardiovascular research (16–20). ML represents a collection of algorithms that autonomously acquire knowledge by identifying patterns from complex, multi-dimensional datasets. ML models can account for interactions between myriads of predictors and their non-linear associations with the outcome; therefore, their utilization could potentially lead to improved explanatory models (21).

In the current study, we sought to implement and evaluate ML algorithms for the prediction of 1- and 3-year all-cause mortality among patients undergoing CRT implantation. We also aimed to explore the sex-specific differences and similarities in the predictors of mortality using advanced ML-based approaches.

## Methods

### Study Population and Protocol

We identified 2,412 patients with chronic HFrEF (NYHA functional class II-IV) who underwent successful CRT implantation at the Heart and Vascular Center of Semmelweis University (Budapest, Hungary) between September 2000 and September 2018. For each patient, pre-implant clinical characteristics (demographics, medical history, physical status, vitals, currently applied medical therapy, ECG-, echocardiographic- and laboratory parameters) and procedural parameters [type of the implanted device, left ventricular (LV) lead position] were collected retrospectively from paper-based or electronic medical records and entered to our structured database.

The study protocol complies with the Declaration of Helsinki, and it was approved by the Regional and Institutional Committee of Science and Research Ethics (Approval No. 161/2019).

### Study Outcomes

Follow-up data [status (dead or alive), date of death] was obtained for all patients by querying the National Health Insurance Database of Hungary in September 2019. Accordingly, all patients included in our database were followed for at least 1 year or died within 1 year. In the entire study population, 2,116 patients also had 3-year outcome data available. The primary endpoint of our study was all-cause mortality.

### Feature Selection and Data Pre-processing

**Figure 1:**
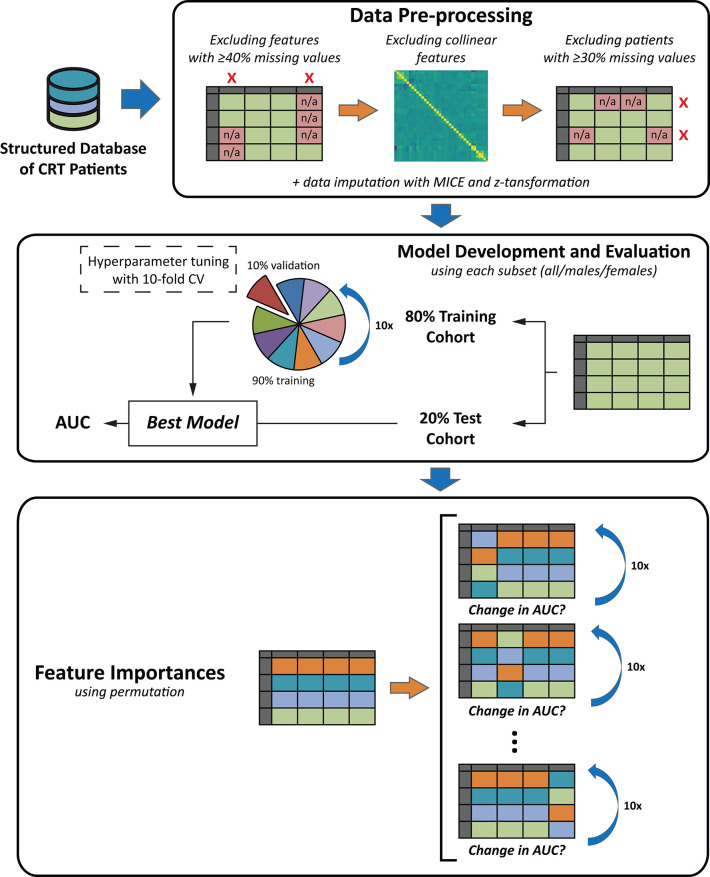
The schematic outline of the data analysis pipeline. The data analysis pipeline included three major steps: (1) data pre-processing, (2) machine learning model development and evaluation, and (3) the calculation of feature importances. During *data pre-processing*, feature selection was performed, patients with a high proportion of missing data were excluded, missing values were imputed using MICE, and z-transformation was performed. Then, *machine learning models* were implemented in the 6 partially overlapping subsets of patients (in all patients, females, or males of the 1- and 3-year cohorts). Before model training, each patient subset was split into training and test cohorts (80:20 ratio). Hyperparameter tuning was performed with 10-fold CV in each training cohort. Models' discriminatory power was estimated using the area under the receiver-operating characteristic curves. Each of the 6 models was retrained in the given training cohort, and its performance was evaluated in the corresponding test cohort. Finally, to identify the most important predictors of mortality in each subset, permutation *feature importances* were computed from each of the 6 final models. See text for further details. AUC, area under the receiver operating characteristic curve; CRT, cardiac resynchronization therapy; CV, cross-validation; MICE, Multiple Imputation by Chained Equations.

The data analysis pipeline, including feature selection, data pre-processing, and ML model development and evaluation is illustrated in Figure 1.

Feature selection included two consecutive steps. First, any feature with ≥40% missing data was removed. Second, collinear variables (Spearman correlation coefficient ≥ 0.3 or ≤-0.3) were also excluded as variables containing redundant information might bias the further steps of the analysis (Supplementary Figure 1). The final set of input features comprised 30 pre-implant and procedural variables: baseline demographics and clinical characteristics (*n* = 10), comorbidities (*n* = 6), ECG- (*n* = 1), laboratory parameters (*n* = 3), and currently applied medications (*n* = 10). The list of candidate variables and the feature selection process are presented in Table 1.

**Table 1:** Steps of feature selection and the list of clinical features included in the machine learning models.

	**Demographics and clinical characteristics**	**Comorbidities**	**ECG**	**Laboratory parameters**	**Medications**
Included in the ML models	Age at CRT implantation Sex Body mass index NYHA functional class HF duration >18 months Etiology of heart failure LVEF LV end-diastolic diameter Type of implanted device LV lead position	Hypertension Diabetes mellitus Type of AFCOPD Smoking status Valvular heart disease	QRS morphology	Hemoglobin Serum sodium Serum creatinine	ACE-I/ARB Beta-blockers CCB Loop diuretics Thiazide diuretics MRA Digitalis Amiodarone Statin Allopurinol
Excluded due to collinearity	Height Weight	History of MI History of CABG and/or PCI		Serum urea GFR	Oral anticoagulants
Excluded due to ≥40% missing values	Systolic blood pressure Diastolic blood pressure Heart rate LV end-diastolic volume LV end-systolic volume		QRS duration PR interval	Lymphocyte Total cholesterol Serum uric acid NT-proBNP	

*Feature selection included two consecutive steps. First, features missing in more than 40% of patients were excluded. Then, collinear variables (Spearman correlation coefficient ≥0.3 or ≤-0.3) were also eliminated as highly correlated variables might bias the further steps of the analysis. The final set of features included 30 clinical variables: age at CRT implantation, sex, body mass index, New York Heart Association functional class, heart failure duration >18 months, etiology of heart failure (ischemic or non-ischemic), left ventricular ejection fraction and end-diastolic diameter assessed with two-dimensional echocardiography, type of the implanted device (CRT-P or CRT-D), left ventricular lead position (anterior, lateral or posterior), hypertension, diabetes mellitus, type of atrial fibrillation (paroxysmal, persistent or permanent), chronic obstructive pulmonary disease, smoking status, valvular heart disease (moderate to severe aortic valve disease, moderate to severe mitral valve disease, severe tricuspid regurgitation), QRS morphology (non-LBBB or LBBB), hemoglobin concentration, serum sodium and creatinine, medical treatment with angiotensin-converting enzyme inhibitors and angiotensin II receptor blockers, beta-blockers, calcium channel blockers, loop diuretics, thiazide diuretics, mineralocorticoid receptor antagonists, digitalis, amiodarone, statins, and allopurinol*.

*ACE-I, angiotensin-converting enzyme inhibitors; AF, atrial fibrillation; ARB, angiotensin II receptor blockers; CABG, coronary artery bypass grafting; CCB, calcium channel blocker; COPD, chronic obstructive pulmonary disease; CRT, cardiac resynchronization therapy; ECG, electrocardiogram; EF, ejection fraction; GFR, glomerular filtration rate; HF, heart failure; LV, left ventricular; MI, myocardial infarction; ML, machine learning; NYHA, New York Heart Association; NT-proBNP, N-terminal pro-brain natriuretic peptide; MRA, mineralocorticoid receptor antagonists; PCI, percutaneous coronary intervention*.

Patients with more than 30% of missing values were excluded from further analyses. Missing values were imputed using Multiple Imputation by Chained Equations (MICE). As the range of different continuous features varied widely, Z-score transformation was applied after imputation to eliminate the possibility of model bias caused by the differing magnitude of the numerical values.

### ML Model Development and Evaluation

We developed ML models to predict two separate outcomes: (1) 1-year all-cause mortality, and (2) 3-year all-cause mortality in the entire cohort, in males and females separately (a total of 6 separate binary classification tasks). To quantify a model's discriminatory power, receiver operating characteristic curve analysis was performed, and the area under the curve (AUC) was calculated. Model development included trials of several binary classifiers such as logistic regression, support vector machines, k-nearest neighbors classifier, gradient boosting classifier, traditional random forest (TRF), conditional inference random forest (CIRF), and multi-layer perceptron.

As the first step of model derivation, 20% of the given patient subset (all, males or females) was randomly selected as the holdout (*test cohort*). This split was performed in a stratified manner to ensure that the original ratio of outcomes is preserved in the training and test cohorts. Hyperparameter tuning was performed with stratified 10-fold cross-validation in the remaining data (80%, *training cohort*). The algorithm (with fine-tuned hyperparameters) exhibiting the highest AUC was then retrained in the entire training cohort, and its performance was evaluated in the test cohort in a statistically independent way. Finally, calibration of the ML models was assessed in the test cohort using Brier score (ranging from 0 to 1, with 0 representing the best possible calibration), which is defined as the mean squared difference between the observed outcomes and the predicted probabilities.

### Feature Importances

To determine the major predictors of 1- and 3-year all-cause mortality in each patient subset, permutation feature importances were computed from each of the 6 final models. Briefly, the importance of an input feature is measured by calculating the increase in the model's prediction error after permuting its values while keeping other features the same as before. In the current study, permutation was performed 10 times for each feature. A feature is considered important if shuffling its values decreases the model's discriminatory power (AUC) as the model relies heavily on that feature for the prediction. On the other hand, a feature is unimportant if shuffling its values leaves the AUC unchanged because, in this case, the model ignores the feature while predicting the outcome. After calculating the importance of each feature, we divided it by the AUC measured in the dataset before shuffling any of its features to enable the comparison of feature importances between different models.

## Results

### Baseline Clinical Characteristics and All-Cause Mortality

**Figure 2:**
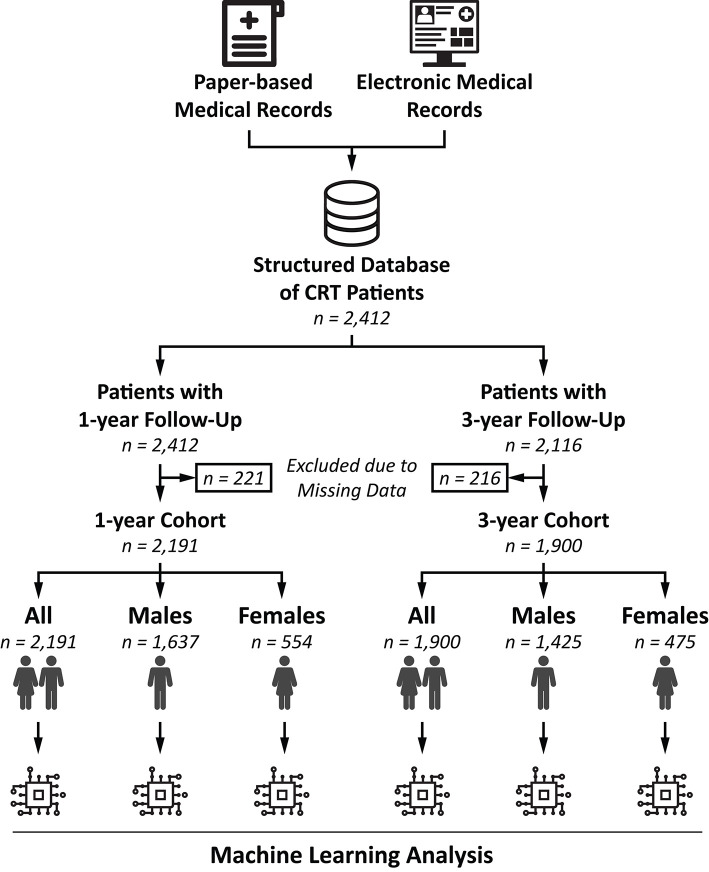
Flowchart illustrating the steps of patient selection. For each patient who underwent successful CRT implantation at our center, pre-implantation clinical characteristics and procedural parameters were collected retrospectively from paper-based or electronic medical records and entered to our structured database. After excluding patients with ≥30% missing values, machine learning models were implemented to predict 1- and 3-year all-cause mortality in the entire cohort, in males and females separately (altogether 6 separate binary classification tasks). CRT, cardiac resynchronization therapy.

The final 1- and 3-year cohorts included 2,191 (74.7% males, 56.7% CRT-D) and 1,900 patients (75.0% males, 54.1% CRT-D), respectively (Figure 2). In the 1-year cohort, 50.4% of the patients had ischemic etiology of HF, 57.8% had NYHA functional class III/IV, and the median left ventricular EF (LVEF) was 28 (24–32) %. In the 3-year cohort, ischemic etiology was reported in 51.5% of the patients, 61.0% presented with NYHA functional class III/IV, and the median LVEF was 28 (24–32) %. The baseline clinical characteristics of the patients are summarized in Tables 2, 3.

**Table 2:** Clinical characteristics of the 1-year cohort.

	**All patients (*n* = 2,191)**	**Males (*n* = 1,637)**	**Females (*n* = 554)**	***p*-value**
**Demographics, vitals, and key electrophysiological characteristics**				
Age, years^*^	68 (61–74)	68 (60–74)	69 (63–75)	<0.001
Weight, kg (1,423)	80 (70–91)	84 (75–95)	70 (60–80)	<0.001
Height, cm (1,413)	172 (165–177)	175 (170–179)	162 (157–167)	<0.001
BMI, kg/m^2^ (1,413)^*^	27.4 (24.5–30.7)	27.6 (24.8–30.8)	26.7 (23.4–30.5)	<0.001
SBP, mmHg (807)	125 (111–136)	125 (111–136)	124 (110–136)	0.403
DBP, mmHg (807)	73 (65–80)	74 (65–80)	71 (64–80)	0.089
NYHA III/IV (1,803)^*^	1,043 (57.8)	781 (57.9)	262 (57.7)	0.945
CRT-D^*^	1,239 (56.5)	1,005 (61.4)	234 (42.2)	<0.001
QRS duration, ms (754)	160 (140–180)	160 (140–180)	160 (140–170)	0.068
QRS morphology, LBBB^*^	1,572 (71.7)	1,127 (68.8)	445 (80.3)	<0.001
LV lead position (1,890)^*^				
Anterior	84 (4.4)	62 (4.4)	22 (4.7)	
Lateral	1,227 (64.9)	932 (65.7)	295 (62.5)	
Posterior	579 (30.6)	424 (25.9)	155 (32.8)	0.442
**Medical history**				
Ischemic etiology of HF^*^	1,104 (50.4)	902 (55.1)	202 (36.5)	<0.001
History of MI	868 (39.6)	713 (43.6)	155 (28.0)	<0.001
HF duration >18 months^*^	680 (31.0)	519 (31.7)	161 (29.1)	0.245
History of or current AF^*^				
No AF	1,394 (63.6)	998 (61.0)	396 (71.5)	
Paroxysmal	342 (15.6)	257 (15.7)	85 (15.3)	
Persistent	59 (2.7)	51 (3.1)	8 (1.4)	
Permanent	396 (18.1)	331 (20.2)	65 (11.7)	<0.001
Valvular heart disease^*^	135 (6.2)	99 (6.0)	36 (6.5)	0.780
Hypertension^*^	1,618 (73.8)	1,216 (74.3)	402 (72.6)	0.459
Diabetes mellitus^*^	813 (37.1)	624 (38.1)	189 (34.1)	0.092
COPD^*^	325 (14.8)	239 (14.6)	86 (15.5)	0.597
Current smoker^*^	131 (6.0)	103 (6.3)	28 (5.1)	0.288
**Laboratory parameters**				
Hemoglobin, g/L (1,440)^*^	136 (123–148)	139 (126–150)	130 (120–140)	<0.001
Serum sodium, mmol/L (1,374)^*^	138 (136–141)	138 (136–140)	139 (136–141)	0.019
Total cholesterol, mmol/L (956)	4.1 (3.4–5.1)	4.0 (3.3–4.9)	4.7 (3.6–5.5)	<0.001
Serum creatinine, μmol/L (1,473)^*^	101 (82–131)	105 (87–134)	86 (71–112)	<0.001
Urea, mmol/L (1,445)	8.3 (6.4–11.7)	8.6 (6.6–11.8)	7.5 (6.0–10.9)	<0.001
Uric acid, μmol/L (766)	405 (322–492)	412 (330–494)	383 (307–474)	0.020
NT-proBNP, pg/mL (309)	2,640 (1,262–3,699)	2,490 (1,367–3,473)	2,680 (1,250–3,710)	0.938
**Echocardiographic parameters**				
LV ejection fraction, % (1,610)^*^	28 (24–32)	28 (23–32)	28 (25–33)	0.046
LVEDD, mL (1,610)^*^	64 (58–70)	65 (59–71)	61 (55–66)	<0.001
**Medications**				
ACE-I/ARB^*^	2,014 (91.9)	1,509 (92.2)	505 (91.2)	0.499
Beta-blocker^*^	1,951 (89.0)	1,457 (89.0)	494 (89.2)	0.914
Ca-channel blocker^*^	127 (5.8)	99 (6.0)	28 (5.1)	0.387
Loop diuretics^*^	1,757 (80.2)	1,315 (80.3)	442 (79.8)	0.780
Thiazide diuretics^*^	516 (23.6)	402 (24.6)	114 (20.6)	0.056
MRA^*^	1,497 (68.3)	1,115 (68.1)	382 (69.0)	0.713
Digitalis^*^	464 (21.2)	359 (21.9)	105 (19.0)	0.138
Amiodarone^*^	593 (27.1)	466 (28.5)	127 (22.9)	0.011
Statin^*^	1,314 (60.0)	995 (60.8)	319 (57.6)	0.184
Allopurinol^*^	591 (27.0)	475 (29.0)	116 (20.9)	<0.001
Oral anticoagulants	729 (33.3)	598 (36.5)	131 (23.6)	<0.001
**Outcome**				
1-year all-cause mortality	252 (11.5)	203 (12.4)	49 (8.8)	0.028

* *Features included in the machine learning models*.

*The value (in parenthesis) after a feature's name indicates the number of patients with available data. If there is no value reported, the given feature was available for all patients*.

*Continuous variables are presented as median (interquartile range), categorical variables as n (%). The comparison between males and females was performed using unpaired Student's t-test or Mann-Whitney U test for continuous variables, Chi-squared or Fisher's exact test for categorical variables, as appropriate*.

*ACE-I, angiotensin-converting enzyme inhibitors; AF, atrial fibrillation; ARB, angiotensin receptor blocker; BMI, body mass index; COPD, chronic obstructive pulmonary disease; CRT-D, cardiac resynchronization therapy defibrillator; DBP, diastolic blood pressure; DM, diabetes mellitus; HF, heart failure; LBBB, left bundle branch block; LVEDD, left ventricular end-diastolic diameter; MI, myocardial infarction; MRA, mineralocorticoid receptor antagonist; NT-proBNP, N-terminal pro-brain natriuretic peptide; NYHA, New York Heart Association functional class; SBP, systolic blood pressure*.

**Table 3:** Clinical characteristics of the 3-year cohort.

	**All patients (*n* = 1,900)**	**Males (*n* = 1,425)**	**Females (*n* = 475)**	***p*-value**
**Demographics, vitals, and key electrophysiological characteristics**				
Age, years^*^	68 (61–74)	68 (60–74)	69 (63–75)	<0.001
Weight, kg (1,280)	80 (70–90)	84 (75–95)	70 (60–80)	<0.001
Height, cm (1,270)	172 (165–177)	175 (170–179)	161 (157–167)	<0.001
BMI, kg/m^2^ (1,270)^*^	27.3 (24.3–30.5)	27.5 (24.7–30.5)	26.5 (23.3–30.5)	<0.001
SBP, mmHg (660)	123 (110–136)	124 (111–136)	122 (110–135)	0.463
DBP, mmHg (660)	72 (65–80)	72 (65–80)	71 (64–80)	0.292
NYHA III/IV (1,568)^*^	956 (61.0)	719 (61.0)	237 (60.9)	0.984
CRT-D^*^	1,027 (54.1)	839 (58.9)	188 (39.6)	<0.001
QRS duration, ms (718)	160 (140–180)	160 (142–180)	160 (140–170)	0.035
QRS morphology, LBBB^*^	1,385 (72.9)	1,000 (70.2)	385 (81.1)	<0.001
LV lead position (1,630)^*^				
Anterior	75 (4.6)	54 (4.4)	21 (5.2)	
Lateral	1,072 (65.8)	814 (66.3)	258 (64.0)	
Posterior	483 (29.6)	359 (29.3)	124 (30.8)	0.633
**Medical history**				
Ischemic etiology^*^	979 (51.5)	802 (56.3)	177 (37.3)	<0.001
History of MI	793 (41.7)	655 (46.0)	138 (29.1)	<0.001
HF duration >18 months^*^	616 (32.4)	477 (33.5)	139 (29.3)	0.090
History of or current AF^*^				
No AF	1,181 (62.2)	850 (59.6)	331 (69.7)	
Paroxysmal	306 (16.1)	227 (15.9)	79 (16.6)	
Persistent	49 (2.6)	43 (3.0)	6 (1.3)	
Permanent	364 (19.2)	305 (21.4)	59 (12.4)	<0.001
Valvular heart disease^*^	131 (6.9)	97 (6.8)	34 (7.2)	0.875
Hypertension^*^	1,417 (74.6)	1,067 (74.9)	350 (73.7)	0.648
Diabetes mellitus^*^	704 (37.1)	542 (38.0)	162 (34.1)	0.125
COPD^*^	288 (15.2)	213 (14.9)	75 (15.8)	0.658
Current smoker^*^	110 (5.8)	89 (6.2)	21 (4.4)	0.140
**Laboratory parameters**				
Hemoglobin, g/L (1,254)^*^	136 (123–148)	139 (125–150)	131 (120–140)	<0.001
Serum sodium, mmol/L (1,180)^*^	138 (136–141)	138 (136–140)	139 (136–141)	0.020
Total cholesterol, mmol/L (827)	4.1 (3.4–5.1)	4 (3.3–4.9)	4.7 (3.6–5.5)	<0.001
Serum creatinine, μmol/L (1,278)^*^	102 (82–132)	106 (87–135)	87 (71–113)	<0.001
Urea, mmol/L (1,254)	8.5 (6.4–11.7)	8.8 (6.6–12.0)	7.7 (6.1–10.9)	<0.001
Uric acid, μmol/L (655)	406 (323–494)	409 (329–495)	386 (313–479)	0.082
NT-proBNP, pg/mL (237)	2,758 (1,398–3,570)	2,610 (1,496–3,376)	2,804 (1,290–3,616)	0.931
**Echocardiographic parameters**				
LV ejection fraction, % (1,378)^*^	28 (24–32)	28 (23–32)	28 (25–32)	0.185
LVEDD, mL (1,378)^*^	64 (58–70)	65 (59–71)	61 (56–67)	<0.001
**Medications**				
ACE-I/ARB^*^	1,731 (91.1)	1,303 (91.4)	428 (90.1)	0.429
Beta-blocker^*^	1,691 (89.0)	1,264 (88.7)	427 (89.9)	0.472
Ca-channel blocker^*^	106 (5.6)	81 (5.7)	25 (5.3)	0.729
Loop diuretics^*^	1,526 (80.3)	1,153 (80.9)	373 (78.5)	0.257
Thiazide diuretics^*^	456 (24.0)	354 (24.8)	102 (21.5)	0.137
MRA^*^	1,270 (66.8)	953 (66.9)	317 (66.7)	0.955
Digitalis^*^	442 (23.3)	341 (23.9)	101 (21.3)	0.234
Amiodarone^*^	528 (27.8)	415 (29.1)	113 (23.8)	0.025
Statin^*^	1,134 (59.7)	862 (60.5)	272 (57.3)	0.214
Allopurinol^*^	521 (27.4)	422 (29.6)	99 (20.8)	<0.001
Oral anticoagulants	627 (33.0)	510 (35.8)	117 (24.6)	<0.001
**Outcome**				
3-year all-cause mortality	615 (32.4)	502 (35.2)	113 (23.8)	<0.001

* *Features included in the machine learning models*.

*The value (in parenthesis) after a feature's name indicates the number of patients with available data. If there is no value reported, the given feature was available for all patients*.

*Continuous variables are presented as median (interquartile range), categorical variables as n (%). The comparison between males and females was performed using unpaired Student's t-test or Mann-Whitney U test for continuous variables, Chi-squared or Fisher's exact test for categorical variables, as appropriate*.

*ACE-I, angiotensin-converting enzyme inhibitors; AF, atrial fibrillation; ARB, angiotensin receptor blocker; BMI, body mass index; COPD, chronic obstructive pulmonary disease; CRT-D, cardiac resynchronization therapy defibrillator; DBP, diastolic blood pressure; DM, diabetes mellitus; HF, heart failure; LBBB, left bundle branch block; LVEDD, left ventricular end-diastolic diameter; MI, myocardial infarction; MRA, mineralocorticoid receptor antagonist; NT-proBNP, N-terminal pro-brain natriuretic peptide; NYHA, New York Heart Association functional class; SBP, systolic blood pressure*.

**Figure 3:**
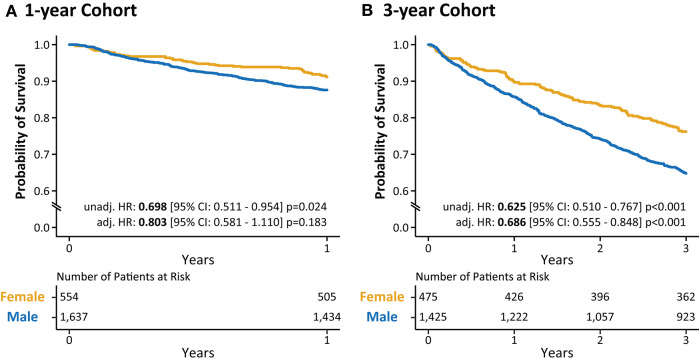
Kaplan-Meier curves for males and females in the 1- **(A)** and 3-year **(B)** cohorts. Kaplan-Meier curve analysis illustrates the difference in the survival of male and female CRT patients during 1- and 3-year follow-up. Cox proportional hazards models were used to compute hazard ratios with 95% confidence intervals. Hazard ratios were adjusted for age (at implantation), QRS morphology, etiology of heart failure, the type of the implanted device, and the type of atrial fibrillation. CI, confidence interval; CRT, cardiac resynchronization therapy; HR, hazard ratio.

In the 1-year cohort, 203 (12.4%) men and 49 (8.8%) women died during the 1-year follow-up period. Univariable Cox regression analysis revealed a significantly lower risk of all-cause mortality in women compared to men [Hazard Ratio (HR): 0.698, 95% Confidence Interval (CI): 0.511–0.954; *p* = 0.024]; however, after adjusting for age, etiology of HF, QRS morphology, type of implanted device, and type of atrial fibrillation (AF, history of or current), we could not observe a significant difference between sexes (HR: 0.803, 95% CI: 0.581–1.110; *p* = 0.183) (Figure 3A).

As observed in the 1-year cohort, males exhibited significantly higher mortality rates compared to females in the 3-year cohort as well [502 (35.2%) vs. 113 (23.8%); *p* < 0.001]. The univariable Cox regression analysis also confirmed this finding as it showed a significantly lower risk of all-cause mortality in females compared to males (HR: 0.625, 95% CI: 0.510–0.767; *p* < 0.001) (Figure 3B). Moreover, this difference remained significant even after adjusting for the previously listed covariates (HR: 0.686, 95% CI: 0.555–0.848; *p* < 0.001).

Patients with ischemic etiology had a significantly increased risk of death in both sexes; however, this difference was more pronounced in females compared to males in the 1- and 3-year cohorts as well (Supplementary Figure 2).

### ML for the Prediction of All-Cause Mortality

Among the evaluated ML classifiers, CIRF exhibited the best performance for discrimination between survival/all-cause death with an AUC of 0.717 (95% CI: 0.676–0.758) and 0.739 (95% CI: 0.715–0.762) in the 1- and 3-year training cohorts, respectively (Supplementary Tables 5, 6). When evaluating the models' discriminatory power in the test cohorts, we observed an AUC of 0.728 (95% CI: 0.645–0.802) and 0.732 (95% CI: 0.681–0.784) for the prediction of 1- and 3-year mortality, respectively. Models were also trained and tested separately in the female and male subsets of the 1- and 3-year cohorts. The AUCs ranged from 0.712 to 0.748 in the training sets and from 0.681 to 0.798 in the test sets suggesting a modest variability in the models' predictive capabilities across the different subsets of patients (Supplementary Tables 7, 8).

After sorting the patients in ascending order based on the predicted probability of death and plotting the distribution of probability values, the accumulation of patients who died during the given follow-up period could be observed in the higher risk regions of the plots (Supplementary Figure 3). These findings suggest that our models can perform risk stratification effectively.

The Brier score—measuring the accuracy of the probabilistic predictions—for the 1- and 3-year models were 0.197 and 0.201, indicating a sufficiently good calibration of our models. Supplementary Table 9 summarizes the Brier scores for the remainder of the CIRF models.

### Most Important Predictors of Mortality as Assessed Using ML

**Figure 4:**
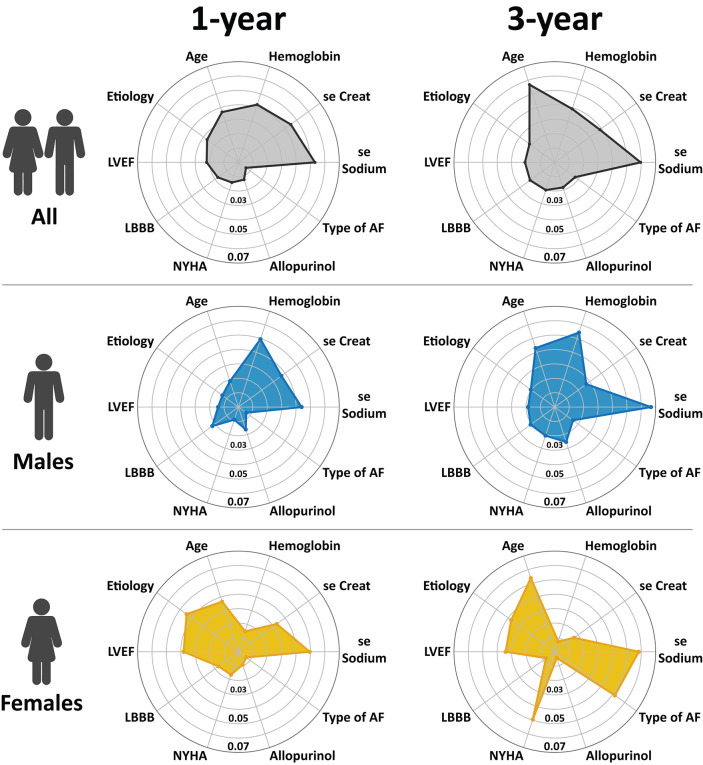
The most important predictors of 1- and 3-year all-cause mortality in patients undergoing CRT implantation. The importance of each feature was quantified with the permutation feature importances method, which measures the importance of a feature by calculating the mean decrease in the model's performance (area under the receiver-operating characteristic curve) after permuting its values 10 times (see text for further details). To keep the data comparable between the different models, we identified the top 5 predictors in each model and took the union of these features; then, we plotted the results on radar charts. AF, atrial fibrillation; LBBB, left bundle branch block; LVEF, left ventricular ejection fraction; NYHA, New York Heart Association.

Leading predictors of all-cause mortality are illustrated in Figure 4, and the comprehensive list of feature importances is provided as Supplementary Tables 10, 11.

#### Top Predictors of Mortality in the 1- and 3-Year Cohorts

In the overall study population (including both sexes), the most important predictor of 1-year mortality was serum sodium, which was followed by serum creatinine, hemoglobin concentration, age, and etiology of HF (Figure 4). These features were also found among the strongest predictors of 3-year mortality, however, in different order of importance (serum sodium, age at implantation, hemoglobin concentration, serum creatinine, and etiology). Digitalis and type of AF were found to show the most prominent change in their importance from 1 to 3 years (both *p* < 0.001).

#### Sex-Specific Patterns of Mortality Predictors at 1-Year Follow-Up

We observed several sex-specific differences during the subgroup analysis. In males, the top predictors of 1-year mortality were hemoglobin concentration, serum sodium, serum creatinine, LBBB morphology, and age, whereas, in females, the most important predictors were serum sodium, etiology, LVEF, age, and serum creatinine (Figure 4).

The comparison of predictors by sex at 1-year revealed that etiology (*p* < 0.001), LVEF (*p* < 0.001), and treatment with amiodarone (*p* < 0.01) were at least twice as important in females as in males. Moreover, age at implantation and NYHA functional class were also significantly more predictive for 1-year mortality in women compared to men (both *p* < 0.001). Whereas, in males, hemoglobin concentration, type of the implanted device, treatment with allopurinol had significantly higher predictive power than in females (all *p* < 0.001).

#### Sex-Specific Patterns of Mortality Predictors at 3-Year Follow-Up

In males, the strongest determinants of 3-year mortality were serum sodium, hemoglobin concentration, age at implantation, serum creatinine, and allopurinol, whereas, in females, these features were serum sodium, age at implantation, type of AF, NYHA functional class, and etiology in decreasing order (Figure 4).

Regarding females, NYHA functional class, etiology, LVEF, and type of AF exhibited significantly higher predictive power than in men (all *p* < 0.001). In males, features with at least a 2-fold higher importance were loop diuretics (*p* < 0.001), hemoglobin concentration (*p* = 0.021), allopurinol (*p* < 0.001), diabetes (*p* < 0.001), LV lead position (*p* < 0.001) and LBBB morphology (*p* < 0.001).

#### Longitudinal Changes in the Sex-Specific Patterns of Mortality Predictors

We also identified features with the most prominent changes in importance from 1 to 3 years of follow-up.

Among males, the most prominent increase of feature importance occurred in LV lead position, NYHA class, age, type of AF, hypertension, and digitalis (all *p* < 0.001). The importance of serum creatinine declined significantly (*p* = 0.026).

In females, we observed the greatest increase in the importance of NYHA functional class (*p* < 0.001), type of AF (*p* < 0.001), hypertension (*p* < 0.001), and age at implantation (*p* < 0.014). Among the top 10 predictors, the most considerable decrease from 1- to 3-year in feature importance was noted in the following factors: serum creatinine, LV end-diastolic diameter, QRS morphology, and amiodarone (all *p* < 0.001).

#### In-depth Analysis of the Associations Between Top Predictors and Outcomes

**Figure 5:**
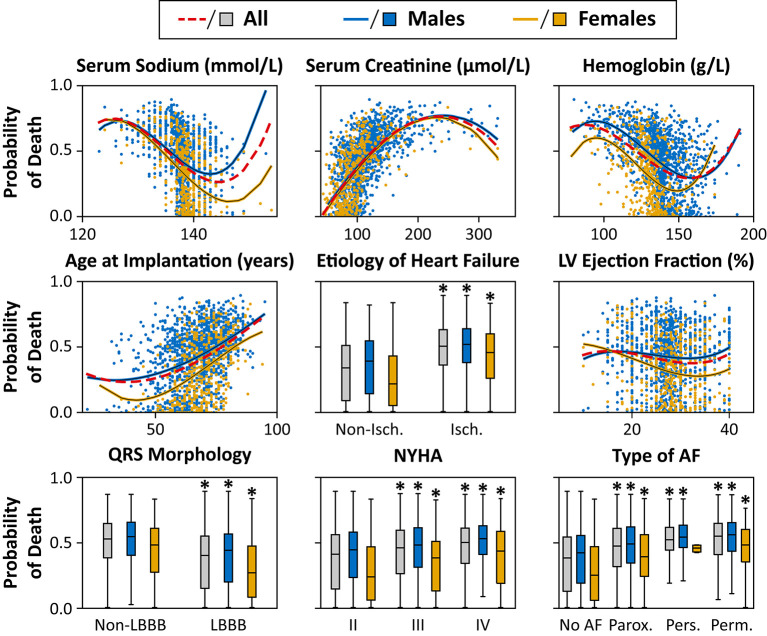
Effect of the most important features on the predicted probability of 1-year all-cause mortality in the training cohorts. The probability of death was calculated for each patient in the training cohort with 10-fold cross-validation. The predicted probability is plotted for each patient, and second-order polynomial trendlines are fitted to their values. *p < 0.05 vs. non-ischemic/non-LBBB morphology/NYHA class II/no AF, unpaired Student's *t*-test or Mann-Whitney *U* test. Abbreviations as in Figure 4.

**Figure 6:**
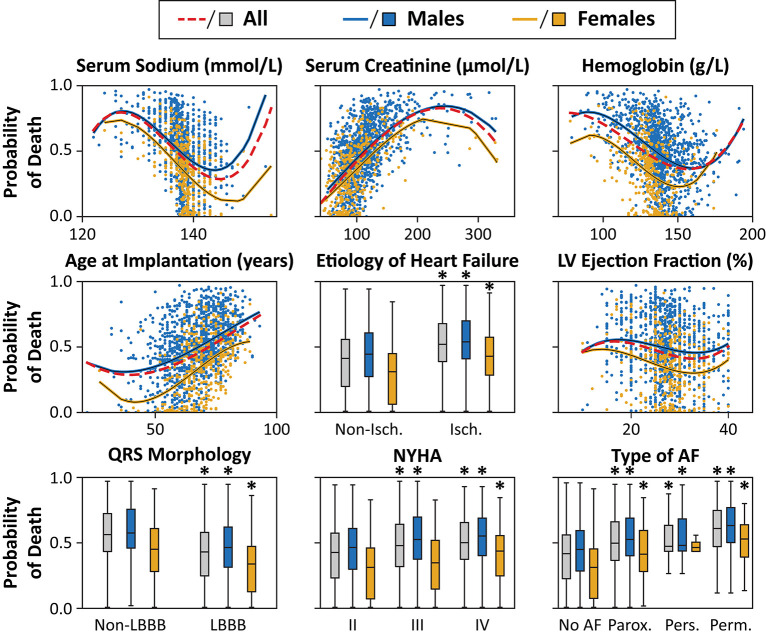
Effect of the most important features on the predicted probability of 3-year all-cause mortality in the training cohorts. The probability of death was calculated for each patient in the training cohort with 10-fold cross-validation. The predicted probabilities are plotted for each patient, and second-order polynomial trendlines are fitted to their values. *p < 0.05 vs. non-ischemic/non-LBBB morphology/ NYHA class II/no AF, unpaired Student's *t*-test or Mann-Whitney *U* test. Abbreviations as in Figure 4.

The association between the most important predictors and the predicted outcome is visually presented in Figures 5, 6. Older age, higher serum levels of creatinine, lower values of LVEF, serum sodium, hemoglobin concentration, ischemic etiology, non-LBBB morphology, higher NYHA classes, and the history of or current paroxysmal, persistent or permanent AF were associated with a higher predicted probability of 1- and 3-year all-cause mortality. Males exhibited higher values of predicted probability of all-cause death in all examined features compared to females. However, as ML models capture complex, high-level interactions among a multitude of variables, it is challenging to determine the effect of a single feature on the predicted probability of mortality, and the results of univariable analyses should be interpreted with caution.

## Discussion

Using data from a single-center cohort of HF patients undergoing CRT implantation, we developed and evaluated ML-based algorithms for the prediction of 1- and 3-year all-cause mortality. The resulting CIRF models demonstrated good discriminatory power in assessing the risk of mortality with an AUC over 0.700 at 1- and 3-year follow-up. Moreover, ML performed substantially well across patient subsets containing exclusively males or females (AUCs ranging from 0.681 to 0.798). Serum sodium, creatinine, hemoglobin, age, and HF etiology were among the most important determinants of short- and mid-term mortality; however, their relative importance varied over time. As expected, female sex was associated with significantly better survival rates in our cohort as well. Sex-specific patterns were also identified in the predictors of mortality. The role of HF etiology (ischemic or non-ischemic), NYHA functional class, and LVEF were more pronounced in females, whereas hemoglobin concentration, QRS morphology, and treatment with allopurinol were notably more predictive for all-cause mortality in males.

### Risk Stratification of HF Patients Using ML

The personalized prediction of prognosis is fundamental to patient-centered care, both in optimizing treatment strategies and informing patients as part of shared decision making. For this purpose, an abundance of prediction models has been developed; however, most of them had achieved only modest success, particularly when they were applied in HF populations other than those from which the scores were derived (22, 23). The unsatisfactory results of previous HF risk scores are likely due to multiple causes, including the fact that most of them were created using conventional statistical methods that failed to capture high-dimensional interactions among predictors that bear relevant prognostic information.

In contrast to traditional statistics, ML was explicitly designed to reveal and harness these correlations. Several studies have proved that these advanced data analytic approaches can leverage the complex, higher-level interplay between predictors and outcomes to achieve better discrimination. ML can improve the care of HF patients in various ways, e.g., by augmenting the prediction of readmission after HF hospitalization or by predicting the risk of mortality (16, 17, 19). In HF patients undergoing CRT implantation, our research group has previously confirmed the superiority of ML over pre-existing risk scores (24), and similar results have been reported by others as well (25, 26). Underpinning these findings, we were able to predict the 1- and 3-year mortality of CRT patients with good discrimination and excellent calibration, even in subsets of patients divided by sex. In light of the promising results of our single-center study, we will endeavor to validate our models in external cohorts in a multi-centric manner.

In our analysis, CIRF exhibited the best discriminative ability for predicting both 1- and 3-year mortality. To understand the outstanding performance of tree-based approaches such as CIRF in outcome prediction, an important difference between conventional regression models and tree-based methods should be highlighted. The former favors variables that have a uniform effect across the entire patient population, whereas the latter can uncover variables that might act differently in different patient subgroups. This is essential for personalized prognostication as in an individual patient, the discriminatory power of a given feature may be significantly enhanced or overshadowed by others. Due to this attribute, tree-based methods such as TRF and CIRF are extremely suitable for application as clinical decision-making tools (27).

### Sex-Specific Differences in Outcomes Following CRT Implantation

Sex is increasingly recognized as an important modulator of outcomes in CRT patients, and several studies such as the MADIT-CRT (10), the RAFT (28), or the MASCOT (29) trials have suggested a greater CRT benefit in women. Despite the expanding knowledge about sex-related differences in HFrEF, the reason women benefit more than men from CRT remains unclear (14). Numerous plausible explanations have been proposed, such as the dissimilarities between sexes in the frequency of ischemic cardiomyopathy (30), AF, and comorbidities (9), or the sex-related differences in body height, LV size, and QRS duration (31, 32). In addition, the impact of sex hormones on the pathophysiology of HF or the sex-specific characteristics of pharmacodynamics and pharmacokinetics are also considerable factors (4, 33).

The sex-specific effects of QRS prolongation and morphology on outcomes have been intensively investigated in CRT patients (30, 31, 34–37). Thus, the findings of these studies have prompted calls for sex-specific guideline recommendations regarding the selection of CRT recipients. As women have shorter QRS durations than men in the absence of any conduction delay, they are more likely to exhibit a true LBBB compared to men at shorter QRS duration (38, 39). It has also been reported that among patients with LBBB and non-ischemic etiology, women have electrical dyssynchrony more frequently compared to men at any given QRS duration, and consequently, they would exhibit a better response to CRT (35). According to the study conducted by Beela et al., the interaction between HF etiology and mechanical dyssynchrony seems to represent another important aspect: due to the lower rate of ischemic etiology and the lower extent of scarred myocardium, women have more frequently uncomplicated patterns of LBBB-like mechanical dyssynchrony which is better amendable by CRT (30).

The beneficial effects of CRT also depend on device programming and the percentage of effective biventricular pacing. Notably, that latter significantly varies by sex, and therefore, sex-specific CRT programming has attracted increased attention (40). According to the results of the SMART-AV trial, the optimization of atrioventricular delay intervals is associated with improved outcomes in women but not in men (41), which might be attributable to the inherent sex-related differences in atrial geometry and PR intervals. A higher percentage of biventricular pacing has also been reported in women (29, 41, 42), most probably due to the lower rate of atrial fibrillation compared to men (43, 44). This could also contribute to the observed differences in mortality between sexes as even a small increment in the biventricular pacing rate may improve outcomes (45).

Although there are still many open questions, it is clear that multiple intercorrelated factors contribute to this phenomenon. Therefore, during the search for answers, ML-based approaches may come in handy, as they are particularly helpful in uncovering hidden patterns in large datasets by simultaneously interpreting predictors even in the presence of complex, non-linear interactions.

### Sex-Specific Patterns in Mortality Predictors

Given the sex-related differences in the anatomy and physiology of the cardiovascular system, encountering dissimilarities in the importance of prognostic predictors between males and females is to be expected in CRT patients. Nevertheless, there is only a limited number of publications dedicated to the thorough exploration of this topic. To the best of our knowledge, our study is the first that evaluated the sex-related differences and similarities in mortality predictors of CRT patients using ML. In our analysis, we observed significant variations in the importance of several predictors such as HF etiology, NYHA functional class, LVEF, and AF between sexes, to name a few.

Utilizing the tools of conventional statistics, the sex-specific prognostic value of HF etiology has been previously investigated in large cohorts of HFrEF patients. In the MAGGIC meta-analysis, the ischemic etiology appeared to attenuate the protective effect of female sex on prognosis (46). In addition, ischemic cardiomyopathy and the extent of myocardial scar were found to be significant predictors of mortality in females but not in males among CRT patients (30). In line with this evidence, the paramount importance of HF etiology in women was proved in our study as well.

When analyzing the interaction between sex and different covariates in the prediction of survival after CRT implantation, Beela et al. reported that NYHA class was a significant predictor in males only (30). Moreover, among HFrEF patients, NYHA class had a more prominent prognostic value in men than in women (3). Contrary to these findings, a stronger association of NYHA functional class with outcomes was observed in females in our current analysis and the BEST trial as well (47).

Another well-established prognostic factor is LVEF, whose interaction with sex in the prediction of all-cause death has been demonstrated in CRT patients (30). Complementing these findings and the results of the BEST trial (47), we have also demonstrated that LVEF is a stronger predictor of prognosis in women than in men.

In HFrEF patients, most studies agree on the prognostic value of AF; however, there is some inconsistency regarding its exact role as some investigations attribute more prognostic impact to AF in females (47), whereas others observed comparable predictive power in males and females (3, 30). Our results support the former as we found AF to have a more prominent effect on outcomes in females.

According to our analysis, the prognostic relevance of hyponatremia and renal function should also be emphasized in CRT patients. Our results are in accordance with the findings of Zusterzeel et al., who reported that despite being significant determinants in both sexes, serum creatinine and hyponatremia appeared to be stronger predictors in women than in men (34).

Lately, the interplay between sex and diabetes in HFrEF patients has attracted increased attention among researchers. Confirming the findings of the MAGGIC (46), the recently published analysis of the ASIAN-HF registry demonstrated that diabetes is coupled with a greater risk of adverse outcomes in women than in men (48). In contrast, diabetes was associated with a higher risk of all-cause death or HF hospitalization in males in the Swedish HF Registry (3), and it was proven to be a significant predictor only in men in the BEST trial (47). Interestingly, in our study, diabetes was not ranked among the top five predictors in any of the analyzed patient subsets, and we detected inter-sex differences in its importance only at 3-year follow-up.

Some of our findings coincide with those of previous studies, whereas some others may not. These apparent discrepancies might be partly attributable to the fact that most studies applied Cox proportional hazards regression, whereas we utilized an entirely different methodology that captures other aspects of associations between risk factors and outcomes. Although the exact reasons behind these contradicting results should be clarified in further investigations, our findings underscore the necessity of sex-specific approaches in the management of HFrEF patients.

### Limitations

Despite the highlighted advantages, there are a few limitations to be acknowledged. First, our study represents results from a single center. As we were aware of this limitation, we performed hyperparameter tuning with 10-fold cross-validation in the training cohorts, and we also tested our models in statistically independent test cohorts to enhance generalizability. Nonetheless, as the next step, the robustness of our models should be tested in external populations as well. Second, the utilized database bears the inherent limitations of retrospective data collection, such as the higher proportion of missing data (compared to prospective trials) and the heterogeneity partly attributable to the changes in guideline recommendations over the years. However, the use of such real-world data holds the potential for better generalizability. Third, our models use baseline (pre-implant and procedural) variables without incorporating the time-varying values of these parameters. Although a dynamic model integrating values of the same parameter from multiple time points may be superior, in the present study, we aimed to predict 1- and 3-year mortality using clinical data that could be acquired at device implantation. Finally, there may remain additional domains of variables (e.g., imaging data, novel biomarkers, genetics, or quality of life questionnaires) that could further improve the predictive capability of our models. Future work should explore the addition of such features to enhance the models proposed in the present study.

## Conclusions

Using advanced ML techniques in combination with easily obtainable clinical features, our models effectively predicted 1- and 3-year all-cause mortality in patients undergoing CRT implantation. ML also exhibited good discriminative ability in patient subsets containing males or females exclusively. Moreover, sex-specific patterns of mortality predictors were identified, which also changed over time. These models lay the foundation stone for future testing of their clinical utility as decision support tools to optimize candidate selection and to improve the prognostication of CRT patients.

## Data Availability Statement

The raw data supporting the conclusions of this article will be made available by the authors, without undue reservation.

## Ethics Statement

The studies involving human participants were reviewed and approved by the Regional and Institutional Committee of Science and Research Ethics (Approval No. 161/2019). Written informed consent for participation was not required for this study in accordance with the national legislation and the institutional requirements.

## Author's Note

A part of the results was presented at the Ph.D. Scientific Days (2020) of the Semmelweis University and at the annual scientific congress of the European Society of Cardiology (ESC Congress 2020—The Digital Experience).

## Author Contributions

MT participated in the conceptualization and designing of the study, implemented the machine learning models and analyzed the data, interpreted the results, and was a major contributor to writing the manuscript. AB was a major contributor to data collection, participated in the interpretation of results, reviewed the literature, and thoroughly reviewed the manuscript. EM participated in data collection, helped in reviewing the literature, and reviewed the manuscript. AKov participated in the conceptualization and designing of the study and critically reviewed the manuscript. ZT made a major contribution to the conceptualization of the study, supervised the machine learning model development, and participated in the interpretation of the results. AS and MC participated in the machine learning model development and helped in the interpretation of the results. BL participated in the interpretation of results, helped in the literature review, and critically reviewed the manuscript. WS contributed to data collection and critically reviewed the manuscript. AKos made a major contribution to the conceptualization of the study and supervised data collection and study execution. BM provided the institutional background of the research, supervised the study execution, and thoroughly reviewed the manuscript. All authors read and approved the final version of the manuscript.

## Conflict of Interest

BM receives lecture fees from Biotronik, Medtronic, and Abbott. ZT is a co-founder and CEO of Argus Cognitive, Inc., holds equity in the company, and receives financial compensation for his work. AS and MC are employees of Argus Cognitive, Inc., and receive compensation for their work. The remaining authors declare that the research was conducted in the absence of any commercial or financial relationships that could be construed as a potential conflict of interest.
