# Supplementary material for: Sex-Specific Patterns of Mortality Predictors Among Patients Undergoing Cardiac Resynchronization Therapy: A Machine Learning Approach
Source: Front Cardiovasc Med. 2021 Feb 25;8:611055. doi: 10.3389/fcvm.2021.611055 (PMC7947699; doi:10.3389/fcvm.2021.611055)
Supplement: Supplementary file 1 [file Data_Sheet_1.docx]

**Sex-Specific Patterns of Mortality Predictors among Patients undergoing Cardiac Resynchronization Therapy: A Machine Learning Approach**

**Supplementary Materials**

**Methods**

***Feature selection***

The excluded features due to collinearity were serum urea (r=0.748 vs. serum creatinine), glomerular filtration rate (r=-0.849 vs. serum creatinine), history of myocardial infarction (r=0.803 vs. etiology of heart failure), history of percutaneous coronary intervention (r=0.675 vs. etiology of heart failure), history of coronary artery bypass grafting (r=0.383 vs. etiology of heart failure), history of ventricular arrhythmia (r=0.313 vs. amiodarone), and oral anticoagulants (r=0.364 vs. type of atrial fibrillation [AF]). As body weight and height also correlated moderately (r=0.582), we used body mass index (BMI) instead.

***Data imputation***

Missing values of continuous variables were imputed using Multiple Imputation by Chained Equations (MICE). This method models each feature with missing values as a function of other features in a round-robin fashion. As opposed to single imputations methods (e.g., mean imputation), it involves multiple imputations in order to mitigate the statistical uncertainty associated with imputations. Missing values of categorical variables were replaced with -1.

***Class imbalance***

To address the class imbalance seen in our database, we assigned more weights to the minority class samples. These weights, in combination with the weighted versions of the machine learning (ML) algorithms, were used during the training process to increase the misclassification cost of minority class samples and to produce models with higher sensitivity and better generalization.

***10-fold cross-validation***

In this procedure, the training cohort is randomly divided into 10 equal folds, each with approximately the same number of events. Ten validation experiments are then performed, with each fold used in rotation as the validation set, and the remaining 9 folds as the training set. Therefore, each data point is used once for testing and 9 times for training, resulting in 10 experimental ML models trained on 90% fractions. The average of results from the 10 experimental models is calculated to provide a measure of the overall performance.

***Traditional random forest (TRF) versus conditional inference random forest (CIRF)***

Like TRF, CIRF is also an ensemble of individual decision trees; however, there is a major difference between the two algorithms: CIRF uses statistical theory (i.e., permutation test) based covariate selection scheme during the training process, whereas TRF creates its trees by selecting the feature that maximizes an information measure (e.g., Gini coefficient) at each split (1,2). Accordingly, the method used in TRF tends to select features that have many possible splits (e.g., continuous or high-cardinality categorical variables); however, CIRF avoids this potential bias and enables the more appropriate calculation of feature importances.

***Conventional statistical analysis***

Continuous variables are expressed as median (interquartile range), while categorical variables are reported as frequencies and percentages. The baseline clinical characteristics of patient subsets were compared with unpaired Student’s t-test or Mann-Whitney U test for continuous variables and Chi-squared or Fisher’s exact test for categorical variables, as appropriate. The survival of subgroups is presented using Kaplan-Meier curves. Cox proportional hazards models were used to compute hazard ratios (HR) with 95% confidence intervals (95% CI). A 2-sided p-value of <0.05 was considered as statistically significant.

***Software packages***

Data preprocessing, feature selection, and ML algorithms were implemented in Python (version 3.6.8, Python Software Foundation, Wilmington, Delaware, U.S.A.) using the scikit-learn module (versions 0.21.3 and 0.23.dev0) (3). Statistical analyses, including group comparisons and survival analyses, were performed in R (version 3.6.1, R Foundation for Statistical Computing, Vienna, Austria) (4).

**Results**

***Baseline clinical characteristics and all-cause mortality***

Female patients were significantly older, were more likely to have non-ischemic etiology and left bundle branch block (LBBB) morphology, and were more frequently implanted with a CRT-P than males in the 1- and 3-year cohorts as well (all p<0.001). Moreover, male patients had significantly higher BMI, were more likely to have a history of or current AF, and had worse renal function compared to females (all p<0.001). Sex-specific differences have also been noted in other laboratory parameters of both cohorts, such as hemoglobin concentration (both p<0.001), serum sodium (p=0.019 and p=0.020), and total cholesterol (both p<0.001). Furthermore, significant differences in the currently applied medical therapy were also observed as men were more frequently treated with amiodarone (p=0.011 and p=0.025), allopurinol (both p<0.001), and oral anticoagulants (p<0.001) compared to women in the 1- and 3-year cohorts as well.

Patients who died during the 1-year follow-up period were older, were more likely to have ischemic etiology and non-LBBB morphology, had lower left ventricular ejection fraction (LVEF), hemoglobin concentration, serum sodium levels, had higher serum creatinine, urea, N-terminal pro-brain natriuretic peptide (NT-proBNP) levels, and were treated with amiodarone more frequently (Supplementary Table 1). After dividing the 1-year cohort based on sex, sex-specific patterns were noted (Supplementary Table 2): LBBB morphology, chronic obstructive pulmonary disease, hemoglobin concentration were associated with death only in males, whereas we found differences in LVEF, amiodarone treatment only between females who were alive at dead at 1-year but not between males.

Patients who died during the 3-year follow-up period were older, were more likely to have ischemic etiology, AF, diabetes mellitus, had lower hemoglobin concentration and serum sodium levels, had higher serum urea, creatinine, and NT-proBNP levels, and were more often treated with digitalis and allopurinol (Supplementary Table 3). The sex-specific analysis revealed that lower values of LVEF were associated with worse outcomes in females but not in males, whereas diabetes mellitus and hemoglobin concentration differed only between males who were dead and alive, but not between the female subgroups (Supplementary Table 4).

The major differences between patients, who survived and died, persisted even after splitting the cohorts into training and test sets (Supplementary Tables 1 and 3).

**References**

1. Breiman L. Random Forests. Machine Learning 2001;45:5-32.

2. Hothorn T, Hornik K, Zeileis A. Unbiased Recursive Partitioning: A Conditional Inference Framework. Journal of Computational and Graphical Statistics 2006;15:651-674.

3. Pedregosa F, Varoquaux G, Gramfort A et al. Scikit-learn: Machine Learning in Python. J Mach Learn Res 2011;12:2825-2830.

4. R Core Team. R: A Language and Environment for Statistical Computing. R Foundation for Statistical Computing, 2017.

**Supplementary Figure 1** Correlation heatmap of the candidate features


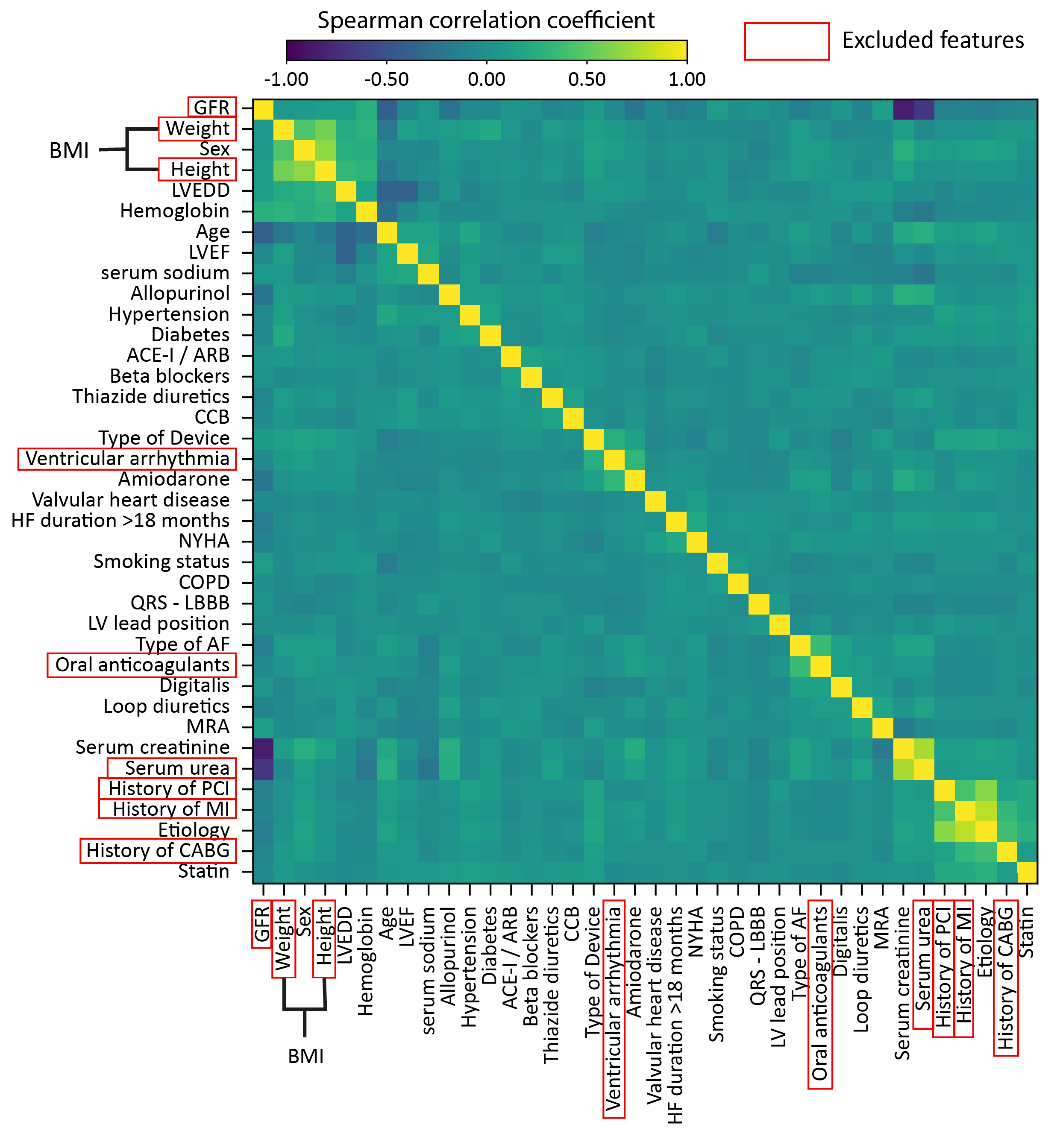
ACE-I – angiotensin-converting enzyme inhibitors, AF – atrial fibrillation, ARB – angiotensin receptor blocker, BMI – body mass index, CABG – coronary artery bypass grafting, CCB – calcium channel blockers, COPD – chronic obstructive pulmonary disease, HF – heart failure, LBBB – left bundle branch block, LVEDD – left ventricular end-diastolic diameter, LVEF – left ventricular ejection fraction, MI – myocardial infarction, MRA – mineralocorticoid receptor antagonist, NYHA – New York Heart Association functional class, PCI – percutaneous coronary intervention

**Supplementary Figure 2** Kaplan-Meier curves for males and females with different etiology of heart failure in the 1- and 3-year cohorts

**
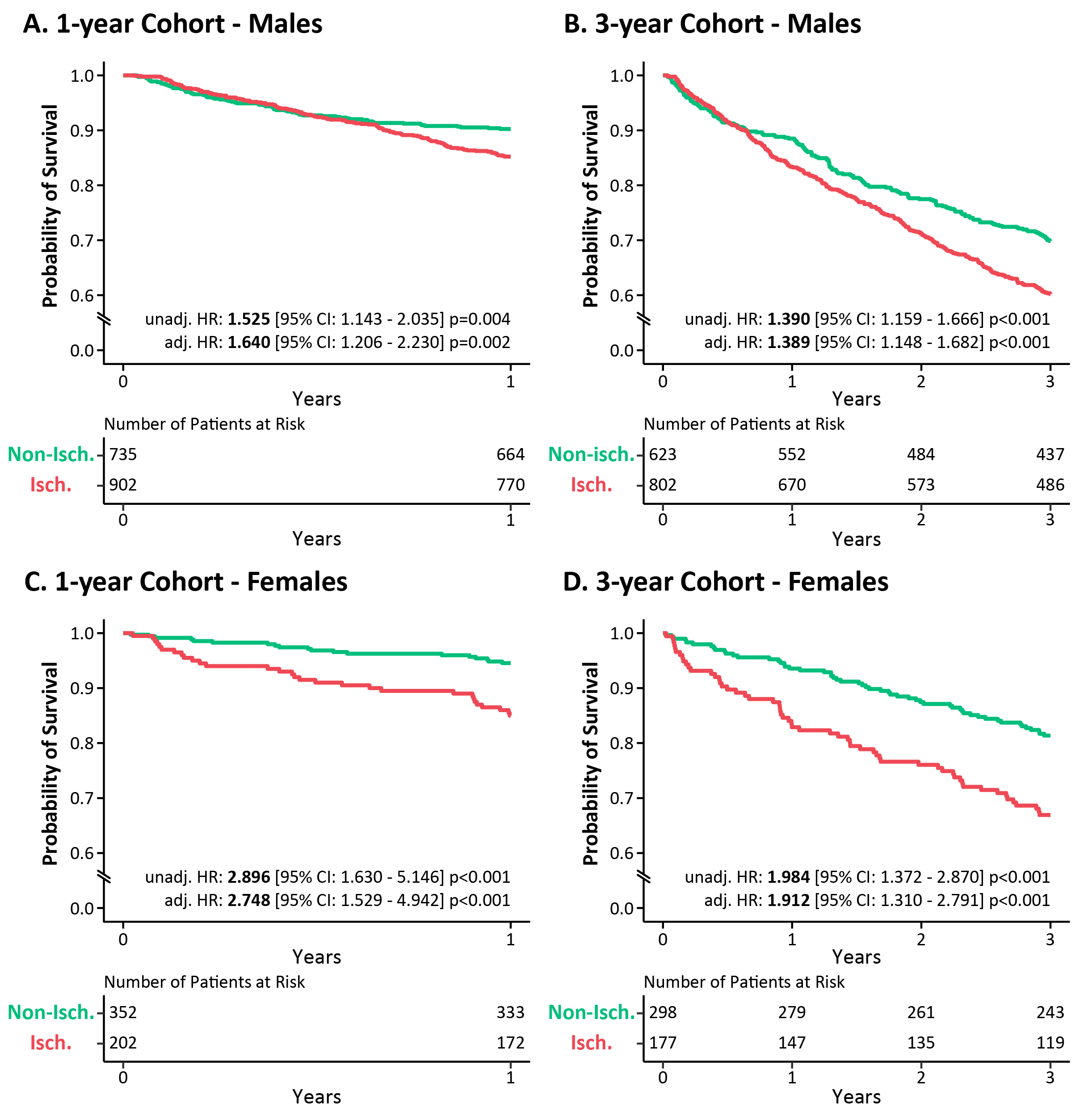
**

Kaplan-Meier curve analysis delineated the sex-specific differences in the survival of CRT patients with different etiology during 1- and 3-year follow-up. Cox proportional hazards models were used to compute hazard ratios with 95% confidence intervals. Hazard ratios were adjusted for age (at implantation), QRS morphology, the type of the implanted device, and the type of atrial fibrillation.

CI – confidence interval, CRT – cardiac resynchronization therapy, HR – hazard ratio

**Supplementary Figure 3** Distribution of the predicted probabilities in the 1- and 3-year test cohorts

**
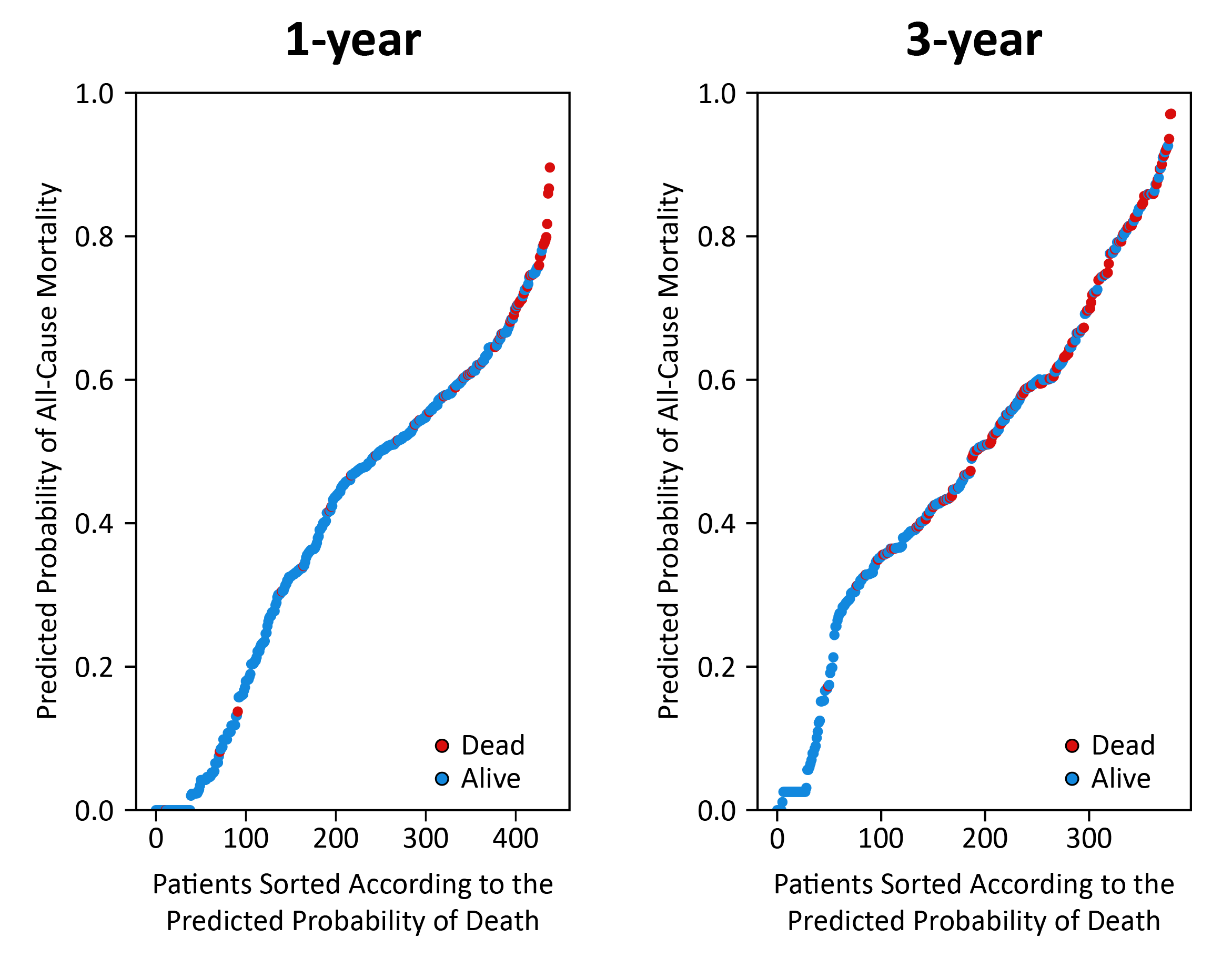
**

Patients were sorted in ascending order based on the predicted probability of death, and the distribution of probability values was plotted. The accumulation of patients who died during the given follow-up period could be observed in the higher risk regions of the plots in the 1- and 3-year cohorts as well. These findings suggest that our models can perform the risk stratification of patients undergoing CRT implantation effectively.

CRT – cardiac resynchronization therapy

**Supplementary Table 1** Clinical characteristics of the 1-year training and test cohorts, comparison of patients alive and dead at 1-year follow-up

|  | **Training Cohort** | | | **Test Cohort** | | |
| --- | --- | --- | --- | --- | --- | --- |
|  | **All (n=1752)** | **Alive (n=1550)** | **Dead (n=202)** | **All (n=439)** | **Alive (n=389)** | **Dead (n=50)** |
| **Demographics, vitals, and key electrophysiological characteristics** | | | | | | |
| Age, years* | 69 (61 - 74) | 68 (61 - 74) | 71 (64 - 77) § | 67 (59 - 74) | 67 (59 - 74) | 67 (62 - 73) |
| Males* | 1299 (74.1) | 1139 (73.5) | 160 (79.2) | 338 (77) | 295 (75.8) | 43 (86.0) |
| Weight, kg (1423) | 80 (70 - 91) | 80 (71 - 92) | 78 (65 - 89) | 80 (70 - 91) | 81 (70 - 92) | 76 (64 - 88) |
| Height, cm (1413) | 172 (165 - 176) | 172 (165 - 177) | 170 (165 - 176) | 172 (165 - 178) | 172 (166 - 178) | 171 (164 - 175) |
| BMI, kg/m2 (1413)* | 27.4  (24.5 - 30.7) | 27.5  (24.7 - 30.7) | 26.3  (23.4 - 30.1) † | 27.4  (24.3 - 30.9) | 27.4  (24.4 - 31.1) | 27.4  (23.7 - 29.4) |
| SBP, mmHg (807) | 125 (111 - 136) | 125 (112 - 137) | 120 (110 - 130) | 125 (110 - 138) | 125 (110 - 138) | 120 (102 - 135) |
| DBP, mmHg (807) | 73 (65 - 80) | 73 (66 - 80) | 71 (61 - 80) | 73 (64.75 - 80) | 74 (65 - 80) | 72 (62 - 77) |
| NYHA III/IV (1803)* | 824 (56.9) | 732 (56.7) | 92 (57.9) | 219 (61.9) | 183 (59.4) | 36 (78.3) † |
| CRT-D* | 986 (56.3) | 884 (57.0) | 102 (50.5) | 253 (57.6) | 224 (57.6) | 29 (58.0) |
| QRS duration, ms (754) | 160 (140 - 180) | 160 (140 - 180) | 160 (140 - 188) | 160 (140 - 180) | 160 (140 - 180) | 160 (145 - 190) |
| QRS morphology, LBBB* | 1274 (72.7) | 1148 (74.1) | 126 (62.4) § | 298 (67.9) | 269 (69.2) | 29 (58.0) |
| LV lead position (1890)* |  |  |  |  |  |  |
| Anterior | 71 (4.7) | 58 (4.3) | 13 (7.6) † | 13 (3.4) | 11 (3.3) | 2 (4.5) |
| Lateral | 980 (64.8) | 862 (64.3) | 118 (68.6) † | 247 (65.3) | 221 (66.2) | 26 (59.1) |
| Posterior | 461 (30.5) | 420 (31.3) | 41 (23.8) † | 118 (31.2) | 102 (30.5) | 16 (36.4) |
| **Medical history** | | | | | | |
| Ischemic etiology* | 895 (51.1) | 766 (49.4) | 129 (63.9) § | 209 (47.6) | 176 (45.2) | 33 (66.0) ‡ |
| History of MI | 708 (40.4) | 605 (39.0) | 103 (51.0) ‡ | 160 (36.4) | 135 (34.7) | 25 (50.0) † |
| HF duration >18 months* | 532 (30.4) | 459 (29.6) | 73 (36.1) | 148 (33.7) | 123 (31.6) | 25 (50.0) † |
| History of or current AF* |  |  |  |  |  |  |
| No AF | 1114 (63.6) | 1001 (64.6) | 113 (55.9) | 280 (63.8) | 249 (64.0) | 31 (62.0) |
| Paroxysmal | 272 (15.5) | 236 (15.2) | 36 (17.8) | 70 (15.9) | 63 (16.2) | 7 (14.0) |
| Persistent | 45 (2.6) | 38 (2.5) | 7 (3.5) | 14 (3.2) | 12 (3.1) | 2 (4.0) |
| Permanent | 321 (18.3) | 275 (17.7) | 46 (22.8) | 75 (17.1) | 65 (16.7) | 10 (20.0) |
| Valvular heart disease* | 106 (6.1) | 87 (5.6) | 19 (9.4) † | 29 (6.6) | 20 (5.1) | 9 (18.0) ‡ |
| Hypertension* | 1305 (74.5) | 1151 (74.3) | 154 (76.2) | 313 (71.3) | 276 (71) | 37 (74.0) |
| Diabetes mellitus* | 641 (36.6) | 559 (36.1) | 82 (40.6) | 172 (39.2) | 150 (38.6) | 22 (44.0) |
| COPD* | 251 (14.3) | 214 (13.8) | 37 (18.3) | 74 (16.9) | 63 (16.2) | 11 (22.0) |
| Current smoker* | 103 (5.9) | 91 (5.9) | 12 (5.9) | 28 (6.4) | 24 (6.2) | 4 (8.0) |
| **Laboratory parameters** | | | | | | |
| Hemoglobin, g/dL (1440)* | 136  (123 - 147) | 137  (124 - 148) | 130  (116 - 143) § | 136  (125 - 150) | 139  (127 - 151) | 129  (112 - 140) § |
| Serum sodium,  mmol/L (1374)* | 138  (136 - 141) | 139  (136 - 141) | 137  (134 - 139) § | 138  (136 - 140) | 139  (136 - 140) | 136  (134 - 139) ‡ |
| Total cholesterol, mmol/L (956) | 4.1 (3.4 – 5.0) | 4.2 (3.4 - 5.0) | 3.7 (3.2 - 4.6) † | 4.2 (3.5 - 5.2) | 4.3 (3.6 - 5.25) | 3.9 (3.4 - 4.9) |
| Serum creatinine, µmol/L (1473)* | 101  (82 - 130) | 99  (80 - 126) | 116  (95 - 154) § | 106  (80 - 132) | 102  (80 - 129) | 119  (102 - 172) § |
| Urea, mmol/L (1445) | 8.3  (6.4 - 11.6) | 8.1  (6.3 - 11.2) | 10.2  (7.6 - 14.7) § | 8.6  (6.4 - 11.9) | 8.3  (6.3 - 11.2) | 10.2  (7.4 - 19.7) ‡ |
| Uric acid, µmol/L (766) | 401  (321 - 487) | 399  (322 - 481) | 423  (313 - 538) | 416  (330 - 506) | 415  (325 - 493) | 512  (360 - 562) † |
| NT-proBNP,  pmol/L (309) | 2756  (1475 - 3706) | 2490  (1266 - 3452) | 3000  (2610 - 4642) ‡ | 2026  (989 - 3343) | 1603  (877 - 2971) | 5341  (3000 - 6559) ‡ |
| **Echocardiographic parameters** | | | | | | |
| LV ejection fraction,  % (1610)* | 28 (24 - 32) | 28 (24 - 32) | 25.5 (22 - 31) † | 28 (24 - 32) | 29 (25 - 33) | 26 (21 - 30) † |
| LVEDD, mm (1610)* | 64 (58 - 70) | 64 (58 - 70) | 64 (59 - 71) | 63 (58 - 69) | 63 (58 - 69) | 62 (57 - 67) |
| **Medications** | | | | | | |
| ACE-I / ARB* | 1605 (91.6) | 1427 (92.1) | 178 (88.1) | 409 (93.2) | 364 (93.6) | 45 (90.0) |
| Beta-blocker* | 1564 (89.3) | 1391 (89.7) | 173 (85.6) | 387 (88.2) | 345 (88.7) | 42 (84.0) |
| Ca-channel blocker* | 101 (5.8) | 88 (5.7) | 13 (6.4) | 26 (5.9) | 26 (6.7) | 0 (0.0) |
| Loop diuretics* | 1403 (80.1) | 1226 (79.1) | 177 (87.6) ‡ | 354 (80.6) | 311 (79.9) | 43 (86.0) |
| Thiazide diuretics* | 428 (24.4) | 379 (24.5) | 49 (24.3) | 88 (20.0) | 78 (20.1) | 10 (20.0) |
| MRA* | 1183 (67.5) | 1052 (67.9) | 131 (64.9) | 314 (71.5) | 279 (71.7) | 35 (70.0) |
| Digitalis* | 372 (21.2) | 326 (21.0) | 46 (22.8) | 92 (21.0) | 75 (19.3) | 17 (34.0) † |
| Amiodarone* | 473 (27) | 404 (26.1) | 69 (34.2) † | 120 (27.3) | 100 (25.7) | 20 (40.0) † |
| Statin* | 1061 (60.6) | 928 (59.9) | 133 (65.8) | 253 (57.6) | 223 (57.3) | 30 (60.0) |
| Allopurinol* | 473 (27) | 396 (25.5) | 77 (38.1) § | 118 (26.9) | 102 (26.2) | 16 (32.0) |
| Oral anticoagulants | 580 (33.1) | 500 (32.3) | 80 (39.6) † | 149 (33.9) | 134 (34.4) | 15 (30.0) |

*Features included in the machine learning models

The value (in parenthesis) after a feature’s name indicates the number of patients with available data. If there is no value reported, the given feature was available for all patients.

Continuous variables are presented as median (interquartile range), categorical variables as n (%).

†p<0.05; ‡p<0.01; §p<0.001 vs. patients alive at 1-year follow-up within the same cohort, unpaired Student’s *t*-test or Mann-Whitney U test for continuous variables, Chi-squared or Fisher’s exact test for categorical variables

ACE-I – angiotensin-converting enzyme inhibitors, AF – atrial fibrillation, ARB – angiotensin receptor blocker, BMI – body mass index, COPD – chronic obstructive pulmonary disease, CRT-D – cardiac resynchronization therapy defibrillator; DBP – diastolic blood pressure, DM, diabetes mellitus; HF – heart failure, LBBB – left bundle branch block, LVEDD – left ventricular end-diastolic diameter, MI – myocardial infarction, MRA – mineralocorticoid receptor antagonist, NT-proBNP – N-terminal pro-brain natriuretic peptide, NYHA – New York Heart Association functional class, SBP – systolic blood pressure

**Supplementary Table 2** Clinical characteristics of males and females in the 1-year cohort, comparison of patients alive and dead at 1-year follow-up

|  | **Males** | | | **Females** | | |
| --- | --- | --- | --- | --- | --- | --- |
|  | **All (n=1637)** | **Alive (n=1434)** | **Dead (n=203)** | **All (n=554)** | **Alive (n=505)** | **Dead (n=49)** |
| **Demographics, vitals, and key electrophysiological characteristics** | | | | | | |
| Age, years* | 68 (60 - 74) | 68 (59.25 - 74) | 70 (62.5 - 75) ‡ | 69 (63 - 75) | 69 (63 - 75) | 70 (65 - 79) † |
| Weight, kg (1423) | 84 (75 - 95) | 84 (75 - 95) | 82 (70 - 92) | 70 (60 - 80) | 70 (60 - 80) | 66 (58 - 75) |
| Height, cm (1413) | 175 (170 - 179) | 175 (170 - 179) | 172 (168 - 178) | 162 (157 - 167) | 162 (157 - 167) | 162 (158 - 165) |
| BMI, kg/m2 (1413)* | 27.6  (24.8 - 30.8) | 27.7  (24.9 - 30.8) | 27.4  (23.9 - 30.1) | 26.7  (23.4 - 30.5) | 26.9  (23.4 - 30.8) | 24.7  (22.3 - 29.4) † |
| SBP, mmHg (807) | 125 (111 - 136) | 125 (112 - 137) | 124 (111 - 133) | 124 (110 - 136) | 125 (110 - 136) | 114 (104 - 130) |
| DBP, mmHg (807) | 74 (65 - 80) | 74 (66 - 80) | 71 (61 - 80) | 71 (64 - 80) | 72 (64 - 80) | 71 (66 - 76) |
| NYHA III/IV (1803)* | 781 (57.9) | 673 (57.0) | 108 (64.3) | 262 (57.7) | 242 (58.0) | 20 (54.1) |
| CRT-D* | 1005 (61.4) | 892 (62.2) | 113 (55.7) | 234 (42.2) | 216 (42.8) | 18 (36.7) |
| QRS duration, ms (754) | 160 (140 - 180) | 160 (140 - 180) | 160 (140 - 180) | 160 (140 - 170) | 160 (140 - 167) | 169 (139 - 200) |
| QRS morphology, LBBB* | 1127 (68.8) | 1007 (70.2) | 120 (59.1) ‡ | 445 (80.3) | 410 (81.2) | 35 (71.4) |
| LV lead position (1890)* |  |  |  |  |  |  |
| Anterior | 62 (4.4) | 48 (3.9) | 14 (7.8) † | 22 (4.7) | 21 (4.8) | 1 (2.7) |
| Lateral | 932 (65.7) | 813 (65.6) | 119 (66.5) † | 295 (62.5) | 270 (62.1) | 25 (67.6) |
| Posterior | 424 (29.9) | 378 (30.5) | 46 (25.7) † | 155 (32.8) | 144 (33.1) | 11 (29.7) |
| **Medical history** | | | | | | |
| Ischemic etiology* | 902 (55.1) | 770 (53.7) | 132 (65) ‡ | 202 (36.5) | 172 (34.1) | 30 (61.2) § |
| History of MI | 713 (43.6) | 609 (42.5) | 104 (51.2) † | 155 (28.0) | 131 (25.9) | 24 (49.0) § |
| HF duration >18 months* | 519 (31.7) | 437 (30.5) | 82 (40.4) ‡ | 161 (29.1) | 145 (28.7) | 16 (32.7) |
| History of or current AF* |  |  |  |  |  |  |
| No AF | 998 (61.0) | 885 (61.7) | 113 (55.7) | 396 (71.5) | 365 (72.2) | 31 (63.3) |
| Paroxysmal | 257 (15.7) | 221 (15.4) | 36 (17.7) | 85 (15.3) | 78 (15.4) | 7 (14.3) |
| Persistent | 51 (3.1) | 43 (3.0) | 8 (3.9) | 8 (1.4) | 7 (1.4) | 1 (2.0) |
| Permanent | 331 (20.2) | 285 (19.9) | 46 (22.7) | 65 (11.7) | 55 (10.9) | 10 (20.4) |
| Valvular heart disease* | 99 (6.0) | 76 (5.3) | 23 (11.3) § | 36 (6.5) | 31 (6.1) | 5 (10.2) |
| Hypertension* | 1216 (74.3) | 1063 (74.1) | 153 (75.4) | 402 (72.6) | 364 (72.1) | 38 (77.6) |
| Diabetes mellitus* | 624 (38.1) | 538 (37.5) | 86 (42.4) | 189 (34.1) | 171 (33.9) | 18 (36.7) |
| COPD* | 239 (14.6) | 197 (13.7) | 42 (20.7) ‡ | 86 (15.5) | 80 (15.8) | 6 (12.2) |
| Current smoker* | 103 (6.3) | 88 (6.1) | 15 (7.4) | 28 (5.1) | 27 (5.3) | 1 (2.0) |
| **Laboratory parameters** | | | | | | |
| Hemoglobin, g/dL (1440)* | 139  (126 - 150) | 140  (128 - 151) | 129  (114 - 144) § | 130  (120 - 140) | 131  119 - 140) | 128  (120 - 135) |
| Serum sodium,  mmol/L (1374)* | 138  (136 - 140) | 138  (136 - 140) | 137  (134 - 140) § | 139  (136 - 141) | 139  (137 - 141) | 136  (135 - 138) § |
| Total cholesterol, mmol/L (956) | 4.0 (3.3 - 4.9) | 4 (3.3 - 4.9) | 3.7 (3.2 - 4.7) † | 4.7 (3.6 - 5.5) | 4.7 (3.7 - 5.5) | 4.0 (3.5 - 4.9) † |
| Serum creatinine, µmol/L (1473)* | 105  (87 - 134) | 103  (85 - 129) | 122  (101 - 161) § | 86  (71 - 112) | 84  (69 - 111) | 106  (86 - 142) § |
| Urea, mmol/L (1445) | 8.6  (6.6 - 11.8) | 8.3  (6.5 - 11.5) | 10.6  (8.0 – 16.0) § | 7.5  (6.0 - 10.9) | 7.5  (5.9 - 10.5) | 7.9  (6.7 - 13.3) |
| Uric acid, µmol/L (766) | 412  (330 - 494) | 410  (331- 492) | 434  (315 - 559) | 383  (307 - 474) | 376  (304 - 464) | 466  (360 - 512) † |
| NT-proBNP,  pmol/L (309) | 2490  (1367 - 3473) | 2462  (1142 - 3413) | 3000  (2856 - 6112) § | 2680  (1250 - 3710) | 2308  (1239 - 3371) | 3000  (2873 - 6480) † |
| **Echocardiographic parameters** | | | | | | |
| LV ejection fraction,  % (1610)* | 28 (23 - 32) | 28 (23 - 32) | 26 (21 - 31) | 28 (25 - 33) | 29 (25 - 33) | 25 (23 - 30) † |
| LVEDD, mm (1610)* | 65 (59 - 71) | 65 (59 - 71) | 64 (59 - 71) | 61 (55 - 66) | 61 (56 - 66) | 62 (55 - 68) |
| **Medications** | | | | | | |
| ACE-I / ARB* | 1509 (92.2) | 1327 (92.5) | 182 (89.7) | 505 (91.2) | 464 (91.9) | 41 (83.7) |
| Beta-blocker* | 1457 (89.0) | 1288 (89.8) | 169 (83.3) ‡ | 494 (89.2) | 448 (88.7) | 46 (93.9) |
| Ca-channel blocker* | 99 (6.0) | 87 (6.1) | 12 (5.9) | 28 (5.1) | 27 (5.3) | 1 (2) |
| Loop diuretics* | 1315 (80.3) | 1138 (79.4) | 177 (87.2) ‡ | 442 (79.8) | 399 (79) | 43 (87.8) |
| Thiazide diuretics* | 402 (24.6) | 349 (24.3) | 53 (26.1) | 114 (20.6) | 108 (21.4) | 6 (12.2) |
| MRA* | 1115 (68.1) | 985 (68.7) | 130 (64.0) | 382 (69.0) | 346 (68.5) | 36 (73.5) |
| Digitalis* | 359 (21.9) | 309 (21.5) | 50 (24.6) | 105 (19.0) | 92 (18.2) | 13 (26.5) |
| Amiodarone* | 466 (28.5) | 397 (27.7) | 69 (34.0) | 127 (22.9) | 107 (21.2) | 20 (40.8) ‡ |
| Statin* | 995 (60.8) | 865 (60.3) | 130 (64.0) | 319 (57.6) | 286 (56.6) | 33 (67.3) |
| Allopurinol* | 475 (29.0) | 396 (27.6) | 79 (38.9) § | 116 (20.9) | 102 (20.2) | 14 (28.6) |
| Oral anticoagulants | 598 (36.5) | 518 (36.1) | 80 (39.4) | 131 (23.6) | 116 (23.0) | 15 (30.6) |

*Features included in the machine learning models

The value (in parenthesis) after a feature’s name indicates the number of patients with available data. If there is no value reported, the given feature was available for all patients.

Continuous variables are presented as median (interquartile range), categorical variables as n (%).

†p<0.05; ‡p<0.01; §p<0.001 vs. patients alive at 1-year follow-up within the same cohort (males or females), unpaired Student’s *t*-test or Mann-Whitney U test for continuous variables, Chi-squared or Fisher’s exact test for categorical variables

ACE-I – angiotensin-converting enzyme inhibitors, AF – atrial fibrillation, ARB – angiotensin receptor blocker, BMI – body mass index, COPD – chronic obstructive pulmonary disease, CRT-D – cardiac resynchronization therapy defibrillator; DBP – diastolic blood pressure, DM, diabetes mellitus; HF – heart failure, LBBB – left bundle branch block, LVEDD – left ventricular end-diastolic diameter, MI – myocardial infarction, MRA – mineralocorticoid receptor antagonist, NT-proBNP – N-terminal pro-brain natriuretic peptide, NYHA – New York Heart Association functional class, SBP – systolic blood pressure

**Supplementary Table 3** Clinical characteristics of the 3-year training and test cohorts, comparison of patients alive and dead at 3-year follow-up

|  | **Training Cohort** | | | **Test Cohort** | | |
| --- | --- | --- | --- | --- | --- | --- |
|  | **All (n=1520)** | **Alive (n=1028)** | **Dead (n=492)** | **All (n=380)** | **Alive (n=257)** | **Dead (n=123)** |
| **Demographics, vitals, and key electrophysiological characteristics** | | | | | | |
| Age, years* | 68 (60 - 74) | 67 (59 - 73) | 70 (63 - 76) § | 68 (62 - 74) | 67 (61 - 74) | 69 (64 - 76) † |
| Males* | 1132 (74.5) | 734 (71.4) | 398 (80.9) § | 293 (77.1) | 189 (73.5) | 104 (84.6) † |
| Weight, kg (1280) | 80 (70 - 90) | 80 (70 - 90) | 80 (70 - 90) | 80 (70 - 90) | 80 (72 - 91) | 79 (67 - 89) |
| Height, cm (1270) | 172 (165 - 176) | 172 (165 - 176) | 171 (165 - 177) | 172 (167 - 178) | 172 (167 - 178) | 172 (165 - 178) |
| BMI, kg/m2 (1270)* | 27.4  (24.4 - 30.7) | 27.6  (24.5 - 30.9) | 26.9  (24.2 - 30.2) | 27.0  (24.2 - 29.8) | 27.1  (24.6 - 30.1) | 26.8  (23.5 - 29.4) |
| SBP, mmHg (660) | 123  (110 - 137) | 125  (114 - 140) | 119  (108 - 132) ‡ | 123  (110 - 133) | 123  (110 - 132) | 122  (111 - 135) |
| DBP, mmHg (660) | 72 (65 - 80) | 73 (65 - 80) | 71 (63 - 80) | 70 (64 - 80) | 70 (64 - 80) | 71 (62 - 78) |
| NYHA III/IV (1568)* | 772 (61.4) | 525 (60.4) | 247 (63.5) | 184 (59.4) | 126 (58.9) | 58 (60.4) |
| CRT-D* | 827 (54.4) | 567 (55.2) | 260 (52.8) | 200 (52.6) | 143 (55.6) | 57 (46.3) |
| QRS duration, ms (718) | 160 (140 - 180) | 160 (140 - 180) | 160 (140 - 186) | 160 (150 - 180) | 160 (149 - 172) | 160 (150 - 189) |
| QRS morphology, LBBB* | 1125 (74.0) | 801 (77.9) | 324 (65.9) § | 260 (68.4) | 181 (70.4) | 79 (64.2) |
| LV lead position (1630)* |  |  |  |  |  |  |
| Anterior | 52 (3.4) | 26 (2.9) | 26 (6.2) ‡ | 23 (6.1) | 17 (7.5) | 6 (5.9) |
| Lateral | 859 (56.5) | 593 (67.2) | 266 (63.5) ‡ | 213 (56.1) | 150 (66.4) | 63 (61.8) |
| Posterior | 391 (25.7) | 264 (29.9) | 127 (30.3) ‡ | 92 (24.2) | 59 (26.1) | 33 (32.4) |
| **Medical history** | | | | | | |
| Ischemic etiology* | 797 (52.4) | 498 (48.4) | 299 (60.8) § | 182 (47.9) | 107 (41.6) | 75 (61.0) § |
| History of MI | 656 (43.2) | 418 (40.7) | 238 (48.4) ‡ | 137 (36.1) | 79 (30.7) | 58 (47.2) ‡ |
| HF duration >18 months* | 489 (32.2) | 313 (30.4) | 176 (35.8) † | 127 (33.4) | 80 (31.1) | 47 (38.2) |
| History of or current AF* |  |  |  |  |  |  |
| No AF | 964 (63.4) | 693 (67.4) | 271 (55.1) § | 217 (57.1) | 155 (60.3) | 62 (50.4) † |
| Paroxysmal | 240 (15.8) | 152 (14.8) | 88 (17.9) § | 66 (17.4) | 44 (17.1) | 22 (17.9) † |
| Persistent | 35 (2.3) | 17 (1.7) | 18 (3.7) § | 14 (3.7) | 11 (4.3) | 3 (2.4) † |
| Permanent | 281 (18.5) | 166 (16.1) | 115 (23.4) § | 83 (21.8) | 47 (18.3) | 36 (29.3) † |
| Valvular heart disease* | 101 (6.6) | 59 (5.7) | 42 (8.5) † | 30 (7.9) | 20 (7.8) | 10 (8.1) |
| Hypertension* | 1139 (74.9) | 771 (75) | 368 (74.8) | 278 (73.2) | 191 (74.3) | 87 (70.7) |
| Diabetes mellitus* | 573 (37.7) | 365 (35.5) | 208 (42.3) † | 131 (34.5) | 75 (29.2) | 56 (45.5) ‡ |
| COPD* | 241 (15.9) | 153 (14.9) | 88 (17.9) | 47 (12.4) | 29 (11.3) | 18 (14.6) |
| Current smoker* | 91 (6.0) | 63 (6.1) | 28 (5.7) | 19 (5.0) | 12 (4.7) | 7 (5.7) |
| **Laboratory parameters** | | | | | | |
| Hemoglobin, g/dL (1254)* | 137  (124 - 148) | 138  (128 - 149) | 133  (119 - 147) § | 136  (121 - 146) | 139  (125 - 147) | 127  (116 - 141) ‡ |
| Serum sodium,  mmol/L (1180)* | 138  (136 - 141) | 139  (137 - 141) | 137  (134 - 140) § | 138  (136 - 140) | 139  (137 - 141) | 137  (134 - 140) § |
| Total cholesterol, mmol/L (827) | 4.1 (3.4 - 5.1) | 4.3 (3.5 - 5.3) | 3.8 (3.2 - 4.7) § | 4 (3.4 - 4.9) | 4.2 (3.6 - 5.1) | 3.8 (3.3 - 4.6) † |
| Serum creatinine, µmol/L (1278)* | 101  (82 - 129) | 95  (78 - 120) | 114  (90 - 149) § | 107  (85 - 141) | 104  (81 - 130) | 114  (92 - 156) ‡ |
| Urea, mmol/L (1254) | 8.4  (6.4 - 11.7) | 7.9  (6.1 - 10.6) | 9.8  (7.2 – 14.0) § | 8.6  (6.8 - 12) | 8.25  (6.5 - 10.7) | 9.3  (7.7 - 14.6) ‡ |
| Uric acid, µmol/L (655) | 408  (326 - 493) | 403  (329 - 484) | 415  (316 - 525) | 401  (301 - 502) | 382  (291 - 464) | 453  (321 - 588) † |
| NT-proBNP,  pmol/L (237) | 2775  (1590 - 3536) | 2254  (1138 - 3000) | 3000  (2331 - 4970) § | 2710  (1164 - 3567) | 1914  (1105 - 3046) | 2921  (1728 - 5148) † |
| **Echocardiographic parameters** | | | | | | |
| LV ejection fraction,  % (1378)* | 28 (24 - 32) | 28 (24 - 32) | 26 (22 - 31) ‡ | 28 (23 - 31) | 28 (24 - 32) | 27 (23 - 30) |
| LVEDD, mm (1378)* | 64 (58 - 70) | 64 (58 - 70) | 65 (59 - 70) | 64 (58 - 71) | 65 (58 - 71) | 62 (58 - 70) |
| **Medications** | | | | | | |
| ACE-I / ARB* | 1386 (91.2) | 948 (92.2) | 438 (89) | 345 (90.8) | 233 (90.7) | 112 (91.1) |
| Beta-blocker* | 1362 (89.6) | 929 (90.4) | 433 (88) | 329 (86.6) | 223 (86.8) | 106 (86.2) |
| Ca-channel blocker* | 88 (5.8) | 66 (6.4) | 22 (4.5) | 18 (4.7) | 12 (4.7) | 6 (4.9) |
| Loop diuretics* | 1229 (80.9) | 798 (77.6) | 431 (87.6) § | 297 (78.2) | 192 (74.7) | 105 (85.4) † |
| Thiazide diuretics* | 357 (23.5) | 243 (23.6) | 114 (23.2) | 99 (26.1) | 60 (23.3) | 39 (31.7) |
| MRA* | 1021 (67.2) | 674 (65.6) | 347 (70.5) | 249 (65.5) | 162 (63.0) | 87 (70.7) |
| Digitalis* | 357 (23.5) | 213 (20.7) | 144 (29.3) § | 85 (22.4) | 55 (21.4) | 30 (24.4) |
| Amiodarone* | 415 (27.3) | 258 (25.1) | 157 (31.9) ‡ | 113 (29.7) | 77 (30.0) | 36 (29.3) |
| Statin* | 916 (60.3) | 618 (60.1) | 298 (60.6) | 218 (57.4) | 141 (54.9) | 77 (62.6) |
| Allopurinol* | 416 (27.4) | 227 (22.1) | 189 (38.4) § | 105 (27.6) | 61 (23.7) | 44 (35.8) † |
| Oral anticoagulants | 506 (33.3) | 320 (31.1) | 186 (37.8) ‡ | 121 (31.8) | 80 (31.1) | 41 (33.3) |

*Features included in the machine learning models

The value (in parenthesis) after a feature’s name indicates the number of patients with available data. If there is no value reported, the given feature was available for all patients.

Continuous variables are presented as median (interquartile range), categorical variables as n (%).

†p<0.05; ‡p<0.01; §p<0.001 vs. patients alive at 3-year follow-up within the same cohort, unpaired Student’s *t*-test or Mann-Whitney U test for continuous variables, Chi-squared or Fisher’s exact test for categorical variables

ACE-I – angiotensin-converting enzyme inhibitors, AF – atrial fibrillation, ARB – angiotensin receptor blocker, BMI – body mass index, COPD – chronic obstructive pulmonary disease, CRT-D – cardiac resynchronization therapy defibrillator; DBP – diastolic blood pressure, DM, diabetes mellitus; HF – heart failure, LBBB – left bundle branch block, LVEDD – left ventricular end-diastolic diameter, MI – myocardial infarction, MRA – mineralocorticoid receptor antagonist, NT-proBNP – N-terminal pro-brain natriuretic peptide, NYHA – New York Heart Association functional class, SBP – systolic blood pressure

**Supplementary Table 4** Clinical characteristics of males and females in the 3-year cohort, comparison of patients alive and dead at 3-year follow-up

|  | **Males** | | | **Females** | | |
| --- | --- | --- | --- | --- | --- | --- |
|  | **All (n=1425)** | **Alive (n=923)** | **Dead (n=502)** | **All (n=475)** | **Alive (n=362)** | **Dead (n=113)** |
| **Demographics, vitals, and key electrophysiological characteristics** | | | | | | |
| Age, years* | 68 (60 - 74) | 67 (59 - 73) | 70 (62 - 76) § | 69 (63 - 75) | 68 (62 - 74) | 71 (66 - 77) § |
| Weight, kg (1280) | 84 (75 - 95) | 84 (75 - 95) | 82 (73 - 94.5) | 70 (60 - 80) | 70 (60 - 80) | 66 (58 - 77) |
| Height, cm (1270) | 175 (170 - 179) | 175 (170 - 178) | 173 (170 - 180) | 161 (157 - 167) | 161 (157 - 167) | 162 (157 - 165) |
| BMI, kg/m2 (1270)* | 27.5  (24.7 - 30.5) | 27.7  (24.8 - 30.8) | 27.4  (24.5 - 30.1) | 26.5  (23.3 - 30.5) | 27.1  (23.0 - 30.6) | 25.7  (22.6 - 29.1) |
| SBP, mmHg (660) | 124  (111 - 136) | 125  (113 - 138) | 122  (110 - 133) † | 122  (110 - 135) | 125  (113 - 139) | 115  (104 - 130) † |
| DBP, mmHg (660) | 72 (65 - 80) | 73 (65 - 80) | 71 (63 - 80) | 71 (64 - 80) | 71 (65 - 80) | 71 (61 - 80) |
| NYHA III/IV (1568)* | 719 (61.0) | 464 (59.9) | 255 (63.1) | 237 (60.9) | 187 (60.7) | 50 (61.7) |
| CRT-D* | 839 (58.9) | 565 (61.2) | 274 (54.6) † | 188 (39.6) | 145 (40.1) | 43 (38.1) |
| QRS duration, ms (718) | 160 (142 - 180) | 160 (142 - 180) | 160 (142 - 190) | 160 (140 - 170) | 160 (140 - 167) | 160 (140 - 180) |
| QRS morphology, LBBB* | 1000 (70.2) | 682 (73.9) | 318 (63.3) § | 385 (81.1) | 300 (82.9) | 85 (75.2) |
| LV lead position (1630)* |  |  |  |  |  |  |
| Anterior | 54 (4.4) | 26 (3.3) | 28 (6.5) † | 21 (5.2) | 17 (5.5) | 4 (4.3) |
| Lateral | 814 (66.3) | 546 (68.3) | 268 (62.6) † | 258 (64.0) | 197 (63.5) | 61 (65.6) |
| Posterior | 359 (29.3) | 227 (28.4) | 132 (30.8) † | 124 (30.8) | 96 (31.0) | 28 (30.1) |
| **Medical history** | | | | | | |
| Ischemic etiology* | 802 (56.3) | 486 (52.7) | 316 (62.9) § | 177 (37.3) | 119 (32.9) | 58 (51.3) § |
| History of MI | 655 (46.0) | 404 (43.8) | 251 (50.0) † | 138 (29.1) | 93 (25.7) | 45 (39.8) ‡ |
| HF duration >18 months* | 477 (33.5) | 290 (31.4) | 187 (37.3) † | 139 (29.3) | 103 (28.5) | 36 (31.9) |
| History of or current AF* |  |  |  |  |  |  |
| No AF | 850 (59.6) | 583 (63.2) | 267 (53.2) ‡ | 331 (69.7) | 265 (73.2) | 66 (58.4) ‡ |
| Paroxysmal | 227 (15.9) | 138 (15.0) | 89 (17.7) ‡ | 79 (16.6) | 58 (16.0) | 21 (18.6) ‡ |
| Persistent | 43 (3.0) | 25 (2.7) | 18 (3.6) ‡ | 6 (1.3) | 3 (0.8) | 3 (2.7) ‡ |
| Permanent | 305 (21.4) | 177 (19.2) | 128 (25.5) ‡ | 59 (12.4) | 36 (9.9) | 23 (20.4) ‡ |
| Valvular heart disease* | 97 (6.8) | 54 (5.9) | 43 (8.6) | 34 (7.2) | 25 (6.9) | 9 (8.0) |
| Hypertension* | 1067 (74.9) | 700 (75.8) | 367 (73.1) | 350 (73.7) | 262 (72.4) | 88 (77.9) |
| Diabetes mellitus* | 542 (38.0) | 320 (34.7) | 222 (44.2) § | 162 (34.1) | 103 (28.5) | 29 (25.7) |
| COPD* | 213 (14.9) | 126 (13.7) | 87 (17.3) | 75 (15.8) | 56 (15.5) | 19 (16.8) |
| Current smoker* | 89 (6.2) | 58 (6.3) | 31 (6.2) | 21 (4.4) | 17 (4.7) | 4 (3.5) |
| **Laboratory parameters** | | | | | | |
| Hemoglobin, g/dL (1254)* | 139  (125 - 150) | 141  (130 - 151) | 132  (117 - 147) § | 131  (120 - 140) | 132  (120 - 140) | 130  (119 - 140) |
| Serum sodium,  mmol/L (1180)* | 138  (136 - 140) | 139  (137 - 141) | 137  (134 - 140) § | 139  (136 - 141) | 139  (137 - 141) | 136  (134 - 138) § |
| Total cholesterol, mmol/L (827) | 4 (3.3 - 4.9) | 4.1 (3.4 - 5) | 3.8 (3.2 - 4.6) § | 4.7 (3.6 - 5.5) | 4.8 (3.77 - 5.6) | 4 (3.2 - 4.9) † |
| Serum creatinine, µmol/L (1278)* | 106  (87 - 135) | 101  (84 - 126) | 116  (94 - 151) § | 87  (71 - 113) | 84  (70 - 109) | 104  (83 - 145) § |
| Urea, mmol/L (1254) | 8.8  (6.6 - 12.0) | 8.1  (6.3 - 10.7) | 9.9  (7.5 - 14.1) § | 7.7  (6.1 - 10.9) | 7.4  (5.9 - 10.4) | 8.8  (6.8 - 13.8) ‡ |
| Uric acid, µmol/L (655) | 409  (329 - 495) | 404  (334 - 485) | 429  (316 - 546) | 386  (313 - 479) | 375  (300 - 463) | 426  (329 - 515) |
| NT-proBNP,  pmol/L (237) | 2610  (1496 - 3376) | 2122  (1097 - 3000) | 3000  (2217 - 4970) § | 2804  (1290 - 3616) | 2404  (1302 - 3282) | 3000  (2499 - 3971) † |
| **Echocardiographic parameters** | | | | | | |
| LV ejection fraction,  % (1378)* | 28 (23 - 32) | 28 (24 - 32) | 28 (22 - 31) | 28 (25 - 32) | 29 (25 - 33) | 25 (23 - 30) ‡ |
| LVEDD, mm (1378)* | 65 (59 - 71) | 65 (59 - 72) | 65 (60 - 71) | 61 (56 - 67) | 61 (56 - 66) | 61 (56 - 67) |
| **Medications** | | | | | | |
| ACE-I / ARB* | 1303 (91.4) | 852 (92.3) | 451 (89.8) | 428 (90.1) | 329 (90.9) | 99 (87.6) |
| Beta-blocker* | 1264 (88.7) | 829 (89.8) | 435 (86.7) | 427 (89.9) | 323 (89.2) | 104 (92.0) |
| Ca-channel blocker* | 81 (5.7) | 57 (6.2) | 24 (4.8) | 25 (5.3) | 21 (5.8) | 4 (3.5) |
| Loop diuretics* | 1153 (80.9) | 712 (77.1) | 441 (87.8) § | 373 (78.5) | 278 (76.8) | 95 (84.1) |
| Thiazide diuretics* | 354 (24.8) | 219 (23.7) | 135 (26.9) | 102 (21.5) | 84 (23.2) | 18 (15.9) |
| MRA* | 953 (66.9) | 598 (64.8) | 355 (70.7) † | 317 (66.7) | 238 (65.7) | 79 (69.9) |
| Digitalis* | 341 (23.9) | 199 (21.6) | 142 (28.3) ‡ | 101 (21.3) | 69 (19.1) | 32 (28.3) † |
| Amiodarone* | 415 (29.1) | 256 (27.7) | 159 (31.7) | 113 (23.8) | 79 (21.8) | 34 (30.1) |
| Statin* | 862 (60.5) | 555 (60.1) | 307 (61.2) | 272 (57.3) | 204 (56.4) | 68 (60.2) |
| Allopurinol* | 422 (29.6) | 221 (23.9) | 201 (40.0) § | 99 (20.8) | 67 (18.5) | 32 (28.3) † |
| Oral anticoagulants | 510 (35.8) | 314 (34.0) | 196 (39.0) | 117 (24.6) | 86 (23.8) | 31 (27.4) |

*Features included in the machine learning models

The value (in parenthesis) after a feature’s name indicates the number of patients with available data. If there is no value reported, the given feature was available for all patients.

Continuous variables are presented as median (interquartile range), categorical variables as n (%).

†p<0.05; ‡p<0.01; §p<0.001 vs. patients alive at 3-year follow-up within the same cohort (males or females), unpaired Student’s *t*-test or Mann-Whitney U test for continuous variables, Chi-squared or Fisher’s exact test for categorical variables

ACE-I – angiotensin-converting enzyme inhibitors, AF – atrial fibrillation, ARB – angiotensin receptor blocker, BMI – body mass index, COPD – chronic obstructive pulmonary disease, CRT-D – cardiac resynchronization therapy defibrillator; DBP – diastolic blood pressure, DM, diabetes mellitus; HF – heart failure, LBBB – left bundle branch block, LVEDD – left ventricular end-diastolic diameter, MI – myocardial infarction, MRA – mineralocorticoid receptor antagonist, NT-proBNP – N-terminal pro-brain natriuretic peptide, NYHA – New York Heart Association functional class, SBP – systolic blood pressure

**Supplementary Table 5** Hyperparameter tuning of different machine learning algorithms in the 1-year cohort

|  | **Hyperparameter Space** | **Best Combination of Hyperparameters** | **AUC in the Training Cohort**  **(10-fold CV)** | **AUC in the Test Cohort** |
| --- | --- | --- | --- | --- |
| **Random Forest** | {‘max_depth’: [1, 2, 4, 5, 10, 25, 40, None], ‘max_features’: [‘log2’, ’sqrt’], ‘min_samples_leaf’: [1, 2, 4], ‘min_samples_split’:[2, 4, 10], ‘n_estimators’: [10, 25, 50, 100, 200, 400]} | {‘max_depth’: 10, ‘max_features’: ‘sqrt’, ‘min_samples_leaf’: 4, ‘min_samples_split’: 2, ‘n_estimator’: 25} | 0.715  [0.675 – 0.755] | 0.701  [0.629 – 0.774] |
| **Conditional Inference Random Forest** | {'alpha': [0.05, 0.1], 'max_depth': [-1, 2, 16], 'min_samples_split': [2, 4, 10], 'n_estimators': [10, 25, 50, 100, 200, 400], 'n_permutations': [50, 100, 150]} | {'alpha': 0.1, 'max_depth': -1, 'min_samples_split': 2, 'n_estimators': 100, 'n_permutations': 50} | **0.717**  [0.676 – 0.758] | 0.728  [0.654 – 0.802] |
| **Logistic Regression (penalty: L1)** | {‘C’: [0.001, 0.01, 0.1, 1, 10, 100], ‘solver’: ['liblinear', 'saga']} | {‘C’: 1,  ‘solver’: liblinear’} | 0.696  [0.654 – 0.737] | 0.720  [0.645 – 0.796] |
| **Logistic Regression (penalty: L2)** | {‘C’: [0.001, 0.01, 0.1, 1, 10, 100], ‘solver’: ['newton-cg', 'lbfgs', 'liblinear', 'sag', 'saga']} | {‘C’: 0.001,  ‘penalty’: ‘l2’,  ‘solver’: ‘liblinear’} | 0.706  [0.664 – 0.747] | 0.725  [0.650 – 0.799] |
| **SVM** | {‘C’: [0.001, 0.01, 0.1, 1, 10, 100],  ‘degree’: [1, 2, 3, 4],  ‘gamma’: [0.1, 1, 10, 100],  ‘kernel’: ['rbf', 'poly']} | {‘C’: 0.001,  ‘degree’: 1,  ‘gamma’: 0. 1,  ‘kernel’: ‘poly’} | 0.692  [0.656 – 0.729] | 0.696  [0.623 – 0.740] |
| **KNN** | {‘algorithm’: ['auto', 'ball_tree','kd_tree','brute'],  ‘leaf_size’: [1, 5, 10, 20, 30, 40, 50],  ‘n_neighbors’: [1, 2, 3, 4, 5, 6, 7, 8, 9, 10, 11, 12, 13, 14, 15, 16, 17, 18, 19, 20, 21, 22, 23, 24, 25],  ‘p’: [1, 2, 3, 4, 5],  ‘weights’: ['uniform', 'distance']} | {‘algorithm’: ‘auto’,  ‘leaf_size’: 1,  ‘n_neighbors’: 23,  ‘p’: 3,  ‘weights’: ‘distance’} | 0.663  [0.633 – 0.694] | 0.662  [0.583 – 0.740] |
| **GBC** | {‘learning_rate’: [1, 0.1, 0.01],  ‘loss’: [‘deviance’],  ‘max_depth’: [1,5,10,20,30,40],  ‘max_features’: [‘log2’,‘sqrt’],  ‘min_smaples_leaf’: [0.1, 0.3, 0.5],  ‘min_samples_split’: [0.1, 0.55, 1],  ‘n_estimators’: [10, 25, 50, 100, 200, 400, 800]} | {‘learning_rate’: 0.01,  ‘loss’: ‘deviance’,  ‘max_depth’: 4,  ‘max_features’: ‘log2’,  ‘min_smaples_leaf’: 0.1,  ‘min_samples_split’: 0.1,  ‘n_estimators’: 400} | 0.700  [0.653 – 0.746] | 0.729  [0.658 – 0.800] |
| **MLP** | {‘activation’: [‘tanh’, ‘relu’],  ‘alpha’: [0.1, 0.01, 0.001, 0.0001],  ‘hidden_layer_sizes’: [(50,), (100,)],  ‘learning_rate’: [‘constant’, ’adaptive’],  ‘solver’: ['sgd', 'adam', 'lbfgs']} | {‘activation’: ‘tanh’,  ‘alpha’: 0.1,  ‘hidden_layer_sizes’: (100,),  ‘learning_rate’: ‘constant’,  ‘solver’: ‘sgd’} | 0.704  [0.663 – 0.746] | 0.728  [0.656 – 0.800] |

Hyperparameters not listed in the table were set to default values predefined in the scikit-learn module (version 0.21.3). Hyperparameter tuning of each machine learning algorithm was performed with grid search. Models with each combination of hyperparameters were trained with stratified 10-fold cross-validation on the training cohort, and the average AUC was calculated. Using the best performing combination of hyperparameters (3rd and 4th columns), models were evaluated in the test cohort as well (5th column). Conditional inference random forest exhibited the best discriminative capability in the training cohort.

AUC – area under the receiver operating characteristic curve, CV – cross-validation, GBC – gradient boosting classifier, KNN – k-nearest neighbors classifier, MLP – multi-layer perceptron, SVM – support vector machines

**Supplementary Table 6** Hyperparameter tuning with different machine learning algorithms in the 3-year cohort

|  | **Hyperparameter space** | **Combination of hyperparameters with the highest AUC** | **AUC in the training cohort** | **AUC in the test cohort** |
| --- | --- | --- | --- | --- |
| **Random Forest** | {‘max_depth’: [1, 2, 4, 5, 10, 25, 40, None], ‘max_features’: [‘log2’, ’sqrt’], ‘min_samples_leaf’: [1, 2, 4], ‘min_samples_split’:[2, 4, 10], ‘n_estimators’: [10, 25, 50, 100, 200, 400]} | {‘max_depth’: 10, ‘max_features’: ‘sqrt’, ‘min_samples_leaf’: 4, ‘min_samples_split’: 10, ‘n_estimator’: 100} | 0.735  [0.713 – 0.758] | 0.716  [0.664 – 0.769] |
| **Conditional Inference Random Forest** | {'alpha': [0.05, 0.1], 'max_depth': [-1, 2, 16], 'min_samples_split': [2, 4, 10], 'n_estimators': [10, 25, 50, 100, 200, 400], 'n_permutations': [50, 100, 150]} | {'alpha': 0.1, 'max_depth': -1, 'min_samples_split': 4, 'n_estimators': 400, 'n_permutations': 100} | **0.739**  [0.715 – 0.762] | 0.732  [0.681 – 0.784] |
| **Logistic Regression (penalty: l1)** | {‘C’: [0.001, 0.01, 0.1, 1, 10, 100], ‘solver’: ['liblinear', 'saga']} | {‘C’: 0.1,  ‘solver’: ‘saga’} | 0.718  [0.695 – 0.741] | 0.720  [0.667 – 0.774] |
| **Logistic Regression (penalty: l2)** | {‘C’: [0.001, 0.01, 0.1, 1, 10, 100], ‘solver’: ['newton-cg', 'lbfgs', 'liblinear', 'sag', 'saga']} | {‘C’: 0.001,  ‘solver’: ‘newton-cg’} | 0.720  [0.696 – 0.744] | 0.724  [0.672 – 0.777] |
| **SVM** | {‘C’: [0.001, 0.01, 0.1, 1, 10, 100],  ‘degree’: [1, 2, 3, 4],  ‘gamma’: [0.1, 1, 10, 100],  ‘kernel’: ['rbf', 'poly']} | {‘C’: 0.01,  ‘degree’: 1,  ‘gamma’: 0. 1,  ‘kernel’: ‘poly’} | 0.717  [0.692 – 0.742] | 0.728  [0.675 – 0.780] |
| **KNN** | {‘algorithm’: ['auto', 'ball_tree','kd_tree','brute'],  ‘leaf_size’: [1, 5, 10, 20, 30, 40, 50],  ‘n_neighbors’: [1, 2, 3, 4, 5, 6, 7, 8, 9, 10, 11, 12, 13, 14, 15, 16, 17, 18, 19, 20, 21, 22, 23, 24, 25],  ‘p’: [1, 2, 3, 4, 5],  ‘weights’: ['uniform', 'distance']} | {‘algorithm’: ‘auto’,  ‘leaf_size’: 1,  ‘n_neighbors’: 22,  ‘p’: 1,  ‘weights’: ‘distance’} | 0.684  [0.661 – 0.706] | 0.658  [0.601 – 0.716] |
| **GBC** | {‘learning_rate’: [1, 0.1, 0.01],  ‘loss’: [‘deviance’],  ‘max_depth’: [1,5,10,20,30,40],  ‘max_features’: [‘log2’,‘sqrt’],  ‘min_smaples_leaf’: [0.1, 0.3, 0.5],  ‘min_samples_split’: [0.1, 0.55, 1],  ‘n_estimators’: [10, 25, 50, 100, 200, 400, 800]} | {‘learning_rate’: 0.1,  ‘loss’: ‘deviance’,  ‘max_depth’: 1,  ‘max_features’: ‘log2’,  ‘min_smaples_leaf’: 0.1,  ‘min_samples_split’: 0.1,  ‘n_estimators’: 400} | 0.736  [0.712 – 0.761] | 0.737  [0.686 – 0.789] |
| **MLP** | {‘activation’: [‘tanh’, ‘relu’],  ‘alpha’: [0.1, 0.01, 0.001, 0.0001],  ‘hidden_layer_sizes’: [(50,), (100,)],  ‘learning_rate’: [‘constant’, ’adaptive’],  ‘solver’: ['sgd', 'adam', 'lbfgs']} | {‘activation’: ‘tanh’,  ‘alpha’: 0.1,  ‘hidden_layer_sizes’: (100,),  ‘learning_rate’: ‘adaptive’,  ‘solver’: ‘sgd’} | 0.718  [0.696 – 0.740] | 0.715  [0.660 – 0.767] |

Hyperparameters not listed in the table were set to default values predefined in the scikit-learn module (version 0.21.3). Hyperparameter tuning of each machine learning algorithm was performed with grid search. Models with each combination of hyperparameters were trained with stratified 10-fold cross-validation on the training cohort, and the average AUC was calculated. Using the best performing combination of hyperparameters (3rd and 4th columns), models were evaluated in the test cohort as well (5th column). Conditional inference random forest exhibited the best discriminative capability in the training cohort.

AUC – area under the receiver operating characteristic curve, CV – cross-validation, GBC – gradient boosting classifier, KNN – k-nearest neighbors classifier, MLP – multi-layer perceptron, SVM – support vector machines

**Supplementary Table 7** Training and testing of the conditional inference random forest models in the entire 1-year cohort (n=2191), males (n=1637), and females (n=554)

|  | **Hyperparameter Space** | **Best Combination of Hyperparameters** | **AUC in the Training Cohort (10-fold CV)** | **AUC in the Test Cohort** |
| --- | --- | --- | --- | --- |
| **Entire 1-year Cohort** | {'alpha': [0.05, 0.1], 'max_depth': [-1, 2, 16], 'min_samples_split': [2, 4, 10], 'n_estimators': [10, 25, 50, 100, 200, 400], 'n_permutations': [50, 100, 150]} | {'alpha': 0.1, 'max_depth': -1, 'min_samples_split': 2, 'n_estimators': 100, 'n_permutations': 50} | 0.717  [0.676 – 0.758] | 0.728  [0.654 – 0.802] |
| **Female** |  | {'alpha': 0.1, 'max_depth': -1, 'min_samples_split': 2, 'n_estimators': 50, 'n_permutations': 50} | 0.748  [0.684 – 0.812] | 0.798  [0.691 – 0.905] |
| **Male** |  | {'alpha': 0.1, 'max_depth': -1, 'min_samples_split': 10, 'n_estimators': 200, 'n_permutations': 50} | 0.712  [0.690 – 0.735] | 0.697  [0.610 – 0.783] |

Hyperparameters not listed in the table were set to default values predefined in the scikit-learn module (version 0.21.3). Hyperparameter tuning was performed with grid search. Each patient subset (entire cohort, males, females) was split into training and test cohorts (80:20 ratio). Models with each combination of hyperparameters were trained with stratified 10-fold cross-validation on these training cohorts, and the average AUC was calculated. Using the best performing combination of hyperparameters (3rd and 4th columns), models were evaluated in the test cohorts of each subset as well (5th column).

AUC – area under the receiver operating characteristic curve, CV – cross-validation

**Supplementary Table 8** Traning and testing of the conditional inference random forest models in the entire 3-year cohort (n=1900), males (n=1425), and females (n=475)

|  | **Hyperparameter Space** | **Best Combination of Hyperparameters** | **AUC in the Training Cohort (10-fold CV)** | **AUC in the Test Cohort** |
| --- | --- | --- | --- | --- |
| **Entire 3-year Cohort** | {'alpha': [0.05, 0.1], 'max_depth': [-1, 2, 16], 'min_samples_split': [2, 4, 10], 'n_estimators': [10, 25, 50, 100, 200, 400], 'n_permutations': [50, 100, 150]} | {'alpha': 0.1, 'max_depth': -1, 'min_samples_split': 4, 'n_estimators': 400, 'n_permutations': 100} | 0.739  [0.715 – 0.762] | 0.732  [0.681 – 0.784] |
| **Female** |  | {'alpha': 0.1, 'max_depth': -1, 'min_samples_split': 10, 'n_estimators': 400, 'n_permutations': 150} | 0.719  [0.678 – 0.760] | 0.777  [0.678 – 0.876] |
| **Male** |  | {'alpha': 0.1, 'max_depth': -1, 'min_samples_split': 4, 'n_estimators': 200, 'n_permutations': 150} | 0.740  [0.717 – 0.763] | 0.681  [0.617 – 0.745] |

Hyperparameters not listed in the table were set to default values predefined in the scikit-learn module (version 0.21.3). Hyperparameter tuning was performed with grid search. Each patient subset (entire cohort, males, females) was split into training and test cohorts (80:20 ratio). Models with each combination of hyperparameters were trained with stratified 10-fold cross-validation on these training cohorts, and the average AUC was calculated. Using the best performing combination of hyperparameters (3rd and 4th columns), models were evaluated in the test cohorts of each subset as well (5th column).

AUC – area under the receiver operating characteristic curve, CV – cross-validation

**Supplementary Table 9** Brier scores of the final machine learning models

|  | **1-year test cohorts** | **3-year test cohorts** |
| --- | --- | --- |
| **All** | 0.197 | 0.201 |
| **Females** | 0.193 | 0.203 |
| **Males** | 0.199 | 0.211 |

Cell contents are Brier scores (calculated using the probabilities of the test cohorts). Brier score is defined as the mean squared difference between the observed and predicted outcome. It ranges from 0 to 1, with values closer to 0 indicating better calibration.

**Supplementary Table 10** Permutation feature importances of the input variables calculated in the 1-year training cohorts

 The importance of each feature was quantified with the permutation feature importances method, which measures the importance of a feature by calculating the mean decrease in the model’s performance (area under the ROC curve) after permuting its values 10 times. Features are sorted according to permutation importance.

ACE-I – angiotensin-converting enzyme inhibitors, AF – atrial fibrillation, ARB - angiotensin II receptor blockers, COPD– chronic obstructive pulmonary disease, CRT – cardiac resynchronization therapy, HF – heart failure, LBBB – left bundle branch block, LV – left ventricular, MRA – mineralocorticoid receptor antagonist, NYHA – New York Heart Association functional class, ROC – receiver operating characteristic

**Supplementary Table 11** Permutation feature importances of the input variables calculated in the 3-year training cohorts

The importance of each feature was quantified with the permutation feature importances method, which measures the importance of a feature by calculating the mean decrease in the model’s performance (area under the ROC curve) after permuting its values 10 times. Features are sorted according to permutation importance.

ACE-I – angiotensin-converting enzyme inhibitors, AF – atrial fibrillation, ARB - angiotensin II receptor blockers, COPD– chronic obstructive pulmonary disease, CRT – cardiac resynchronization therapy, HF – heart failure, LBBB – left bundle branch block, LV – left ventricular, MRA – mineralocorticoid receptor antagonist, NYHA – New York Heart Association functional class, ROC – receiver operating characteristic
